# Succinylation heterogeneity in lung adenocarcinoma: from prognostic model to KLK6-driven tumor microenvironment remodeling

**DOI:** 10.3389/fimmu.2025.1718994

**Published:** 2025-11-26

**Authors:** Jichang Liu, Xuehan Zhu, Chenlong Zha, Jiaqi Ding, Chuanpeng Zhang, Yizhe Wang, Tao Yan, Hui Kong, Yong Liu, Jingyu Chen

**Affiliations:** 1 Lung Transplantation Center, The Affiliated Wuxi People’s Hospital of Nanjing Medical University, Wuxi, Jiangsu, China; 2 Department of Pulmonary & Critical Care Medicine, The First Affiliated Hospital of Nanjing Medical University, Nanjing, Jiangsu, China; 3 Department of Thoracic Surgery, Affiliated Hospital of Qingdao University, Qingdao, Shandong, China; 4 Department of Lung Transplantation, The Second Affiliated Hospital Zhejiang University School of Medicine, Hangzhou, Zhejiang, China

**Keywords:** succinylation, lung adenocarcinoma, KLK6, prognosis, immunotherapy

## Abstract

**Background:**

Lung adenocarcinoma (LUAD) is a leading cause of cancer-related mortality. Protein succinylation, a key post-translational modification, is implicated in tumor progression. However, its comprehensive landscape and clinical significance in LUAD remain largely unexplored.

**Methods:**

We integrated multi-omics data from The Cancer Genome Atlas (TCGA) and Gene Expression Omnibus (GEO) cohorts. A set of core succinylation-related genes was identified through differential expression and univariable Cox regression analyses. Molecular subtypes based on succinylation were determined by principal component analysis (PCA). A succinylation prognostic model was constructed via least absolute shrinkage and selection operator (LASSO) and multivariable Cox regression. The differences of tumor microenvironment (TME), tumor mutation burden and drug sensitivity in different risk groups were further explored. Single-cell RNA sequencing (scRNA-seq) and spatial transcriptomics revealed effects of succinylation on TME. High-dimensional weighted gene co-expression networks analysis (hdWGCNA) was used to identify potential succinylation-related therapeutic targets. The function of therapeutic targets was further validated through scRNA-seq, spatial transcriptomics, and *in vitro* experiments.

**Results:**

We identified 31 core succinylation-related genes and defined three molecular subtypes with distinct prognostic and TME characteristics. A robust 7-gene succinylation-based prognostic signature was developed and validated across 7 independent GEO cohorts, effectively stratifying patients into high- and low-risk groups with significant differences in survival, demonstrating high predictive accuracy, consistency, and clinical utility. The low-risk group exhibited an immunoreactive TME with enhanced immune cell infiltration and superior response to immunotherapy. scRNA-seq and spatial transcriptomics revealed enhanced succinylation in LUAD. Kallikrein-related peptidase 6 (KLK6) was identified as a potential therapeutic target. KLK6 was significantly upregulated in LUAD, correlated with poor prognosis and therapy resistance. KLK6 promoted global succinylation, proliferation, migration, and invasion of LUAD cells *in vitro*. Mechanistically, KLK6-positive tumor cells might foster an immunosuppressive TME by driving fibroblast-to-myofibroblast differentiation, enhancing extracellular matrix (ECM) deposition, and inhibiting CD8^+^ T cell infiltration.

**Conclusion:**

Our study delineates the succinylation landscape in LUAD, establishes a novel prognostic model for risk stratification and immunotherapy prediction. Meanwhile, we identified KLK6 as a potential promoter of tumor progression and immunosuppression. Targeting the succinylation pathway, particularly KLK6, may represent a promising therapeutic strategy for LUAD.

## 1 Introduction

Lung adenocarcinoma (LUAD), the most prevalent subtype of non-small cell lung cancer (NSCLC), accounts for approximately 50% of all lung cancer cases globally and represents a leading cause of cancer-related mortality (1, 2). Over recent decades, substantial progress has been made in the detection, diagnosis, and management of LUAD, particularly with the advent of immune checkpoint inhibitors (ICIs), which have markedly improved clinical outcomes in eligible patients (3). Despite these therapeutic advances, long-term survival rates remain unsatisfactory, likely due to the complex crosstalk between tumor cells and the immune microenvironment (4). Consequently, there is a compelling need to delineate the molecular mechanisms underlying poor prognosis in LUAD and to establish reliable molecular stratification frameworks that can guide both prognostic evaluation and personalized immunotherapeutic strategies.

Mitochondrial dysfunction and metabolic reprogramming are hallmarks of cancer, yet the role of post-translational modifications (PTMs) in these processes remains underexplored (5, 6). Succinylation, a widespread PTM involving the addition of a succinyl group to lysine residues, has recently gained attention for its ability to regulate enzyme activity and metabolic pathways (7, 8). Succinyl-coenzyme A (succinyl-CoA), a key intermediate in the tricarboxylic acid (TCA) cycle, serves as the substrate for succinylation, linking this modification directly to cellular energy metabolism (9). In LUAD, mitochondrial dysfunction often manifests as altered succinylation patterns, which can influence tumor proliferation and survival (7). For instance, succinyl-coenzyme A synthetase GDP-forming subunit β (SUCLG2) has been shown to regulate global succinylation levels in LUAD cells, and its loss promotes tumorigenesis by destabilizing metabolic enzymes (7). Similarly, crosstalk between succinylation and other PTMs, such as phosphorylation and acetylation, adds layers of complexity to cancer signaling networks (10, 11). Moreover, succinylation can specifically modulate immune responses. It has been reported that 3-Oxoacid CoA-Transferase 1 (OXCT1) promotes hepatocellular progression via succinylating Lactamase Beta (LACTB) (12). Succinylation-mediated regulation of immune checkpoint molecules impacts immunotherapy outcomes (13, 14). Recent proteomic studies have identified succinylation as a potential biomarker in LUAD (15). For example, succinylome profiling of non-smoking female LUAD patients revealed 2,373 succinylation sites on 1,205 proteins, with upregulated succinylation enriched in mitochondrial metabolic pathways (15). However, a systematic analysis of succinylation-related genes and their clinical implications in LUAD is lacking. Moreover, the interplay between succinylation, the tumor immune microenvironment, and spatial architecture remains poorly understood.

In this study, we integrated multi-omics data to map the succinylation landscape of LUAD. Firstly, we identified 31 core succinylation-related genes by differential expression profiling and univariable Cox regression analysis. Next, we defined three distinct succinylation-related molecular subtypes, and constructed a prognostic model validated across 7 independent GEO cohorts. The model stratified LUAD patients into high- and low-risk groups and could predict prognosis and immunotherapy response accurately. tumor microenvironment (TME) heterogeneity, Tumor Mutation Burden (TMB), tumor stemness and drug sensitivity between different risk groups were also revealed. Using single-cell and spatial transcriptomics, we dissect how succinylation influences TME remodeling and immunotherapy response. Finally, we nominate KLK6 as a succinylation-associated oncogene that promotes LUAD progression through spatial coordination of stromal and immune cells and validated the effects of KLK6 on LUAD proliferation, migration, invasion, succinylation, and fibroblast-to-myofibroblast differentiation *in vitro*. KLK6-positive tumor cells might foster an immunosuppressive TME by driving fibroblast-to-myofibroblast differentiation, enhancing extracellular matrix (ECM) deposition, and inhibiting CD8^+^ T cell infiltration. Our work reveals the heterogeneity of succinylation in LUAD and highlights potential of KLK6 as a therapeutic target of LUAD.

## 2 Materials and methods

### 2.1 Data collection and processing

Gene expression data for lung adenocarcinoma (LUAD) and clinical information were obtained from The Cancer Genome Atlas (TCGA) (https://cancergenome.nih.gov/) and Gene-Expression Omnibus (GEO) (https://www.ncbi.nlm.nih.gov/geo/) databases. After excluding patients with missing prognostic data or zero survival time, 500 LUAD cases from TCGA database and 1115 LUAD cases from GEO database were retrieved as the training cohort and validation cohort, respectively (GSE3141: 58 cases; GSE13213: 117 cases; GSE30219: 83cases; GSE31210: 226 cases; GSE37745: 106; GSE50081: 127 cases; GSE72094: 398 cases). Demographic characteristics were summarized in **Supplementary Table S1**. To mitigate batch effects across GEO datasets, we applied the ComBat algorithm from the ‘sva’ R package (16). Somatic mutation and copy number variation (CNV) data for TCGA-LUAD were also extracted from TCGA. A total of 615 succinylation-related genes were identified from the GeneCards database (https://www.genecards.org/).

To validate the predictive efficacy of the risk model for immunotherapy response, we analyzed seven independent immunotherapeutic cohorts: (1)advanced urothelial cancer treated with atezolizumab, an anti-Programmed Cell Death Ligand 1 (PD-L1) antibody (IMvigor210 cohort) (17); (2) melanoma treated with anti-Cytotoxic T Lymphocyte-Associated Antigen-4 (CTLA4) and anti-Programmed Cell Death 1 (PD-1) therapy (GSE91061) (18); (3) metastatic melanoma treated with anti-CTLA4 therapy (19); (4) NSCLC treated with nivolumab or pembrolizumab, an anti-PD-1 antibody (GSE126044) (20); (5) NSCLC treated with anti-PD-1/PD-L1 antibody (GSE135222) (21); (6) melanoma treated with adoptive T cell therapy (ACT) (GSE100797) (22); (7) Melanoma treated with anti-PD-1 antibody (GSE78220). The response and benefit of TCGA cohort were calculated based on the Tumor immune dysfunction and exclusion (TIDE) website (http://tide.dfci.harvard.edu/), which integrated TIDE score, interferon gamma (INFG), microsatellite instability (MSI) score, CD274, Merck18, CD8, myeloid-derived suppressor cells (MDSC), cancer associated fibroblast (CAF) and tumor-associated macrophages (TAM) M2.

Single-cell RNA sequencing (scRNA-seq) data comprising LUAD and normal lung tissue samples were obtained from Code Ocean (DOI: 10.24433/CO.0121060.v1) (23). The GSE207442 dataset provided additional immunotherapy response data for NSCLC (24). Spatial transcriptomics data were obtained from 10x Genomics Visium datasets (E-MTAB-13530 and GSE189487) (25).

### 2.2 Identification of core succinylation-related genes

To identify core succinylation-related genes, differential expression analysis was performed using the TCGA LUAD dataset to screen for differentially expressed succinylation-related genes (False discovery rate, FDR < 0.05 & |log2fold Change| > 1). Subsequently, univariable Cox regression analysis was applied to further identify succinylation-related genes with prognostic value (p < 0.05). The intersection of prognostic succinylation-related genes from the TCGA and GEO datasets yielded the final core succinylation-related genes. CNV of these core succinylation-related genes in LUAD were then analyzed. Using these core genes, we calculated a succinylation score for each sample via single-sample Gene Set Enrichment Analysis (ssGSEA). Next, we compared succinylation scores between LUAD and normal lung tissues, and overall survival (OS) differences between patients with high- and low-succinylation scores. The optimal cutoff value was determined by the ‘survminer’ package.

### 2.3 Principal Component Analysis (PCA)

To characterize succinylation heterogeneity in LUAD, PCA was employed to stratify LUAD patients into three molecularly distinct clusters based on 31 core succinylation-related genes. Both survival outcomes and succinylation scores were compared across these subgroups. A clinical correlations heatmap was visualized. To elucidate the biological implications of succinylation modification, we conducted comprehensive functional enrichment analyses. Gene Ontology (GO) and Kyoto Encyclopedia of Genes and Genomes (KEGG) pathway analyses were used to identify differentially regulated biological processes and Hallmark gene set analysis was used to characterize distinct biological signatures among the three clusters. Infiltration of various immune cells was also compared among the three clusters.

### 2.4 Construction and validation of a succinylation-related prognostic model

We developed a prognostic model through the following steps: (1) identifying common differentially expressed genes (DEGs) shared among all clusters (p < 0.001); (2) screening DEGs with univariable Cox regression (p < 0.05); (3) further screening DEGs via Least Absolute Shrinkage and Selection Operator (LASSO) regression; and (4) building a succinylation-related prognostic model by multivariable Cox regression analysis. A risk score was calculated for each patient based on the model. The importance of each gene in the model was evaluated using SHAP (SHapley Additive exPlanations) analysis. Patients with LUAD were stratified into high-risk and low-risk groups based on the optimal risk score cutoff. Kaplan–Meier survival analysis was performed to compare survival outcomes between the two groups in both the training and validation cohorts. The predictive accuracy of the model was evaluated using time-dependent receiver operating characteristic (ROC) curves generated via the “timeROC” package with the “Aalen” weighting approach. Furthermore, the model’s ability to predict immunotherapy response was validated across seven independent immunotherapy cohorts.

The prognostic significance of clinical characteristics and the risk scores was assessed by univariable and multivariable Cox regression analyses. The independent prognostic factors were integrated to construct a predictive nomogram using the “rms” R package. The nomogram was evaluated by ROC analysis, calibration curves, and decision curve analysis (DCA).

### 2.5 TME landscape analyses

Based on the ESTIMATE algorithm, we computed the immune score, stromal score, ESTIMATE score, and tumor purity for each sample. The infiltration levels and functional states of immune cells were further assessed using ssGSEA. We compared the expression of immune checkpoint genes and HLA-related genes between the high- and low-risk groups. Additionally, the scores representing various steps of the cancer-immunity cycle for TCGA-LUAD samples were obtained from the TIP database (http://biocc.hrbmu.edu.cn/TIP/) (26) and were compared between the high- and low-risk groups.

### 2.6 TMB and drug sensitivity analyses

Somatic mutation profiles were analyzed with the “maftools” R package. TMB was compared between the high- and low-risk groups. To assess the combined prognostic effect of TMB and risk stratification, patients were classified into four groups based on median TMB values and the level of risk score for Kaplan–Meier survival analysis. Furthermore, drug sensitivity analysis for 137 compounds was conducted in different risk groups using the “pRRophetic” R package, which employs ridge regression to estimate half-maximal inhibitory concentration (IC_50_) values (27). The results were visualized as a parliament plot by the Hiplot Pro platform (https://hiplot.com.cn).

### 2.7 scRNA-seq dataset analysis and cell annotation

scRNA-seq data were processed with Seurat (v5.3.0). Quality control was performed by filtering out cells with fewer than 300 or more than 7,000 genes, those with more than 100,000 transcripts, cells with greater than 20% mitochondrial gene content, and cells with more than 5% hemoglobin gene expression. PCA was conducted on highly variable genes to reduce the dimensionality by the “RunPCA” function. Then, Cell neighborhoods were constructed with the “FindNeighbors” function, and clustering was conducted using “FindClusters”. Cells were annotated based on the established marker gene expression profiles. Cell subpopulations were visualized in two-dimensional space using Uniform Manifold Approximation and Projection (UMAP) or t-distributed Stochastic Neighbor Embedding (t-SNE).

To distinguish malignant from non-malignant cells, copy number karyotyping was performed using Copy number karyotyping of aneuploid tumors (CopyKAT) under default parameters (28). This tool estimates genomic copy number variations at an average 5 Mb resolution based on scRNA-seq data. Tumor cells were identified by aneuploid genomic profiles, whereas normal cells were characterized by diploid copy number spectra.

The succinylation activity of individual cells was evaluated using multiple scoring algorithms: AUCell (v1.30.0), UCell (v2.12.0), SingScore, ssGSEA, and AddModuleScore. Each method generated a normalized score reflecting succinylation activity. The final activity score was derived from the integrated output of these approaches.

### 2.8 High-dimensional weighted gene co-expression networks analysis (hdWGCNA)

To precisely identify genes significantly associated with malignant cell subpopulations exhibiting distinct succinylation activity levels, we implemented an analytical pipeline leveraging hdWGCNA for scRNA-seq data (29). Malignant cells were first stratified based on their succinylation scores using a graph-based clustering algorithm grounded in shared nearest neighbor (SNN) principles. Subsequently, hdWGCNA was applied to uncover highly correlated gene modules. Genes in key modules with strong co-expression patterns were selected for downstream analyses.

### 2.9 Pseudotime trajectory, transcription factor analysis and cell-cell interaction analysis

The differentiation trajectory for tumor cells was inferred using the R package “Monocle” (v2.32.0) and “Monocle3” (v1.4.26). Single-cell regulatory network inference and clustering (SCENIC) is a new computational method to map genes regulatory networks and identify stable cell states by evaluating the activity of each cell from scRNA-seq data (30). In this study, we utilized the SCENIC to identify key transcription factors (TFs) associated with succinylation heterogeneity in tumor cells. Intercellular communication networks were deciphered using the R package “CellChat”. Receptor-ligand interactions were quantified with computeCommunProb() at a minimum probability threshold of 0.05 to exclude weak signals.

### 2.10 Analysis of spatial transcriptomics data for LUAD

Spatial data (10x Visium) were processed with Seurat. Quality control excluded ribosomal and mitochondrial genes. Data were normalized with SCTransform. Dimensionality reduction was carried out via PCA, followed by UMAP for visualization. Cell types were annotated using Robust Cell Type Decomposition (RCTD) by integrating a reference scRNA-seq dataset (DOI: 10.24433/CO.0121060.v1). The standard RCTD workflow was rigorously applied with the doublet mode configured to “full” to enhance accuracy in cell type assignment within spatially resolved spots (31). To quantify the influence of cellular spatial organization on intercellular interactions, we employed the MISTy framework implemented in the mistyR package (v1.2.1), considering intra-spot (intrinsic) and inter-spot (para, radius=6) views. Additionally, spatially variable genes and receptor-ligand interactions were systematically analyzed using SpaGene to identify localized signaling patterns within the tissue architecture.

### 2.11 Cell culture and transfections

A549 and MRC-5 cells (Procell, Wuhan, China) were respectively cultured in Ham’s F-12K (Kaighn’s) and dulbecco’s modified eagle medium, DMEM (Procell, Wuhan, China), supplemented with 10% fetal bovine serum (FBS) in a humidified atmosphere of 5% CO2 and 37°C according to protocol. KLK6 siRNAs (Gene&Bio Co. Ltd, Shandong, China) and plasmid (Gene&Bio Co. Ltd, Shandong, China) were transfected into cells using Lipofectamine^®^ 3000 (Invitrogen) according to the manufacturer’s instructions. The siRNA duplex sense sequence was as follows: siKLK6: 5 “-GCAAGACAGCAGAUGGUGATT-3”.

### 2.12 RNA extracting and real-time PCR

Total RNA was extracted from LUAD cells using the RNAeasyTM kit (R0026; Beyotime Biotechnology) following the manufacturer’s instructions. The mRNA (500ng) was reverse-transcribed into complementary DNA (cDNA) using the HiScript III First Strand cDNA Synthesis Kit (+gDNA wiper) (Vazyme Biotech Co., Ltd., China). Then, cDNA was amplified with SYBR Premix kit (Vazyme, Co., Ltd China). The mRNA levels were assayed by qRT-PCR using the Applied Biosystems qPCR system (Thermo Fisher Scientific, Waltham, MA, USA). Relative quantification of target gene expression was determined using the 2^-ΔΔCt^ method, with GAPDH serving as the endogenous reference gene for normalizatio. The sequences of the primers were listed in **Supplementary Table S2**.

### 2.13 Western blot

Total cellular proteins were extracted using RIPA lysis buffer supplemented with protease and phosphatase inhibitors. Protein concentration was determined using a BCA protein assay kit (Beyotime, Shanghai, China) according to the manufacturer’s instructions. Equal amounts of protein samples were separated by SDS-polyacrylamide gel electrophoresis (SDS-PAGE) and subsequently transferred onto a polyvinylidene fluoride (PVDF) membrane (Millipore, Billerica, USA). QuickBlock blocking buffer (Beyotime, Shanghai, China) was used to blocking and then the membrane was incubated with primary antibody at 4 °C overnight. The primary antibodies were listed as follows: KLK6, GAPDH (Proteintech, USA, 1:1000); ACTA2, FN1 (CST, USA, 1:1000); anti-succinyllysine antibody (PTM BioLab, Cat#PTM-419, 1:1000). After washing with TBST solution, the membrane was incubated with corresponding horseradish peroxidase (HRP)-labeled secondary antibody (Proteintech, USA, 1:10000) for 1h. The ECL kit and FluorChem E system were used for detection (Proteinsimple, CA, USA).

### 2.14 Immunohistochemical staining

A formalin-fixed, paraffin-embedded (FFPE) tissue array was purchased from Shanghai OUTDO BIOTECH Co.,Ltd and used for IHC analysis. Array was deparaffinized in xylene and rehydrated through a graded series of ethanol solutions. Endogenous peroxidase activity was quenched by incubation in 3% hydrogen peroxide for 15 minutes. Antigen retrieval was performed using citrate-EDTA buffer (Beyotime, Shanghai, China) at 96–98°C for 15 minutes. Primary antibodies, including anti-KLK6 (1:200; Proteintech, USA), were applied to the sections and incubated overnight at 4°C. After washing, the sections were incubated with HRP-conjugated anti-rabbit secondary antibody for 30 minutes at 37°C, followed by amplification with HRP-labeled streptavidin for an additional 30 minutes. Two independent pathologists, blinded to the experimental conditions, evaluated the immunostained sections. Staining intensity was scored on a scale of 0 to 3: 0: no staining; 1: light yellow; 2: light brown; 3: deep brown. The percentage of positive cells was categorized as follows: 0: <5%; 1: 5–25%; 2: 26–50%; 3: 51–75%; 4: >75%. The final IHC score was calculated by multiplying the intensity score by the proportion score, yielding a total range of 0 to 12.

### 2.15 Cell proliferation assay

A cell proliferation assay was conducted using a Cell Counting Kit-8 (CCK-8; HY-K0301, MCE) according to the manufacturer’s instructions. Briefly, cells were seeded into 96-well plates at a density of 1,000 cells per well in triplicate. After incubation for 24, 48, 72, and 96 hours under standard culture conditions, 10 ;μL of CCK-8 solution was added to each well and incubated for 1 hour at 37°C. The absorbance of each well was measured at a wavelength of 450 ;nm using a microplate reader (Skanlt RE 7.0; Thermo Scientific, USA).

### 2.16 Wound-healing assay

Cells were seeded in 6-well plates and cultured until reaching 80–90% confluence. A sterile pipette tip was used to generate a straight scratch wound uniformly across the cell monolayer. The dislodged cells and debris were gently removed by washing with phosphate-buffered saline (PBS). The culture medium with 2% fetal bovine serum was added. The scratch area was captured every 12 hours and the migration rate were measured by Image J.

### 2.17 Transwell experiment

Cell invasion and migration capacities were assessed using Transwell chambers with 8-μm pores (Corning Inc., NY, USA). For the invasion assay, inserts were pre-coated with Matrigel^®^ basement membrane matrix (Corning Inc.) to simulate extracellular matrix conditions. For the migration assay, uncoated Transwell inserts were used in parallel. Cells were resuspended in serum-free medium and seeded into the upper chamber. The lower chamber was filled with medium supplemented with 20% FBS as a chemoattractant. After incubation for 24–48 hours at 37 °C, cells that had invaded or migrated to the lower surface were fixed with methanol, stained with 0.2% crystal violet, and imaged under a light microscope. Cell counts were used as the mean ± standard error, an unpaired T test was used for comparisons between different groups, and a two-tailed p value < 0.05 was considered statistically significant.

### 2.18 Immunofluorescence staining

Cells were plated on glass coverslips in 24-well plates. Cells were fixed with ice-cold 4% Paraformaldehyde for 20 min and permeabilized with 0.2% Triton X-100 for 10 min. Then cells were washed with PBS, blocked in 10% goat serum albumin for 30 min. Anti-ACTA2 (CST, USA: 1:200), anti-COL1A1 (CST, USA, 1:200) were primary antibodies. Cells were incubated by the primary antibodies at 4°C overnight. Next, cells were rinsed with PBS and incubated with Alexa Fluor 594 and Alexa Fluor 488 for 1h at room temperature in the dark and counterstained with DAPI (Beyotime, Shanghai, China), mounted and examined using a fluorescence microscope (Olympus, Tokyo, Japan). The colocalization was analyzed by image J.

A formalin-fixed, paraffin-embedded (FFPE) tissue array was purchased from Shanghai OUTDO BIOTECH Co.,Ltd and used for multiple immunofluorescence staining. Anti-KLK6 (Proteintech, USA, 1:200), anti-ACTA2 (CST, USA: 1:200), anti-CD8A (CST, USA: 1:200) were primary antibodies.

### 2.19 Cell co-culture assay

An indirect co-culture system was established using Transwell inserts (Corning, USA) with 0.4 μm porous membranes. MRC-5 cells were seeded into the lower compartment of a 6-well plate in complete medium and allowed to adhere for 4 hours under standard culture conditions (37°C, 5% CO_2_). Subsequently, tumor cells were seeded onto the Transwell membrane. The co-culture system was maintained for 24–48 hours. Then, RNA or protein of MRC-5 cells was extracted for the further study.

### 2.20 Statistical analyses

All statistical analyses were performed using R software (v4.5.1), GraphPad Prism 8 and SPSS v26. Differences in continuous variables between two independent groups were assessed using the Wilcoxon rank-sum test, while comparisons among more than two groups were conducted with the Kruskal-Wallis test. For categorical variables, group differences were evaluated using chi-square test. Correlations between continuous variables were examined using Spearman’s rank correlation. A two-sided p-value of less than 0.05 was considered statistically significant. FDR was used to adjust p-value.

## 3 Results

### 3.1 Identification of core succinylation-related genes

**Figure 1:**
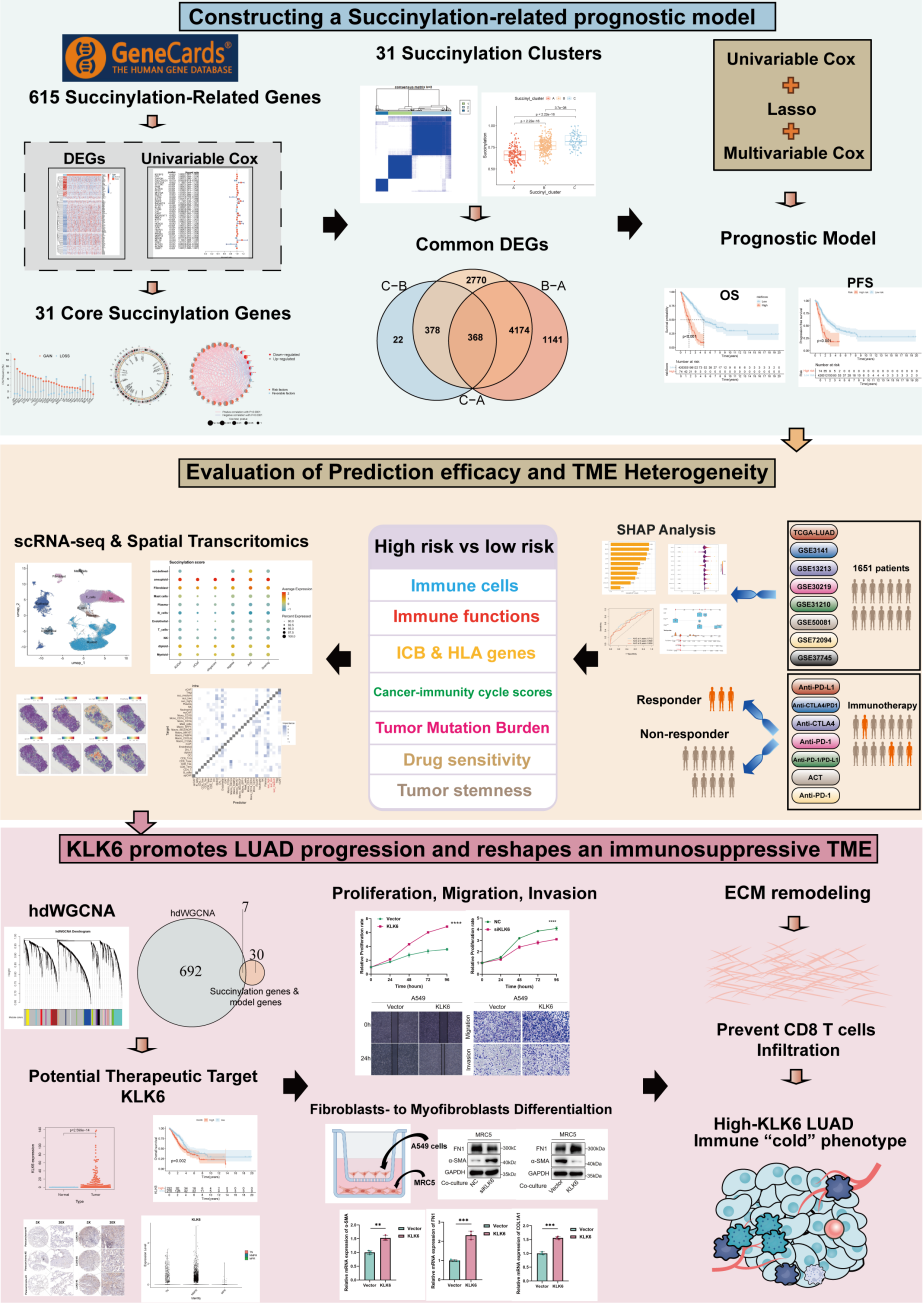
The flow chart of the study.

**Figure 2:**
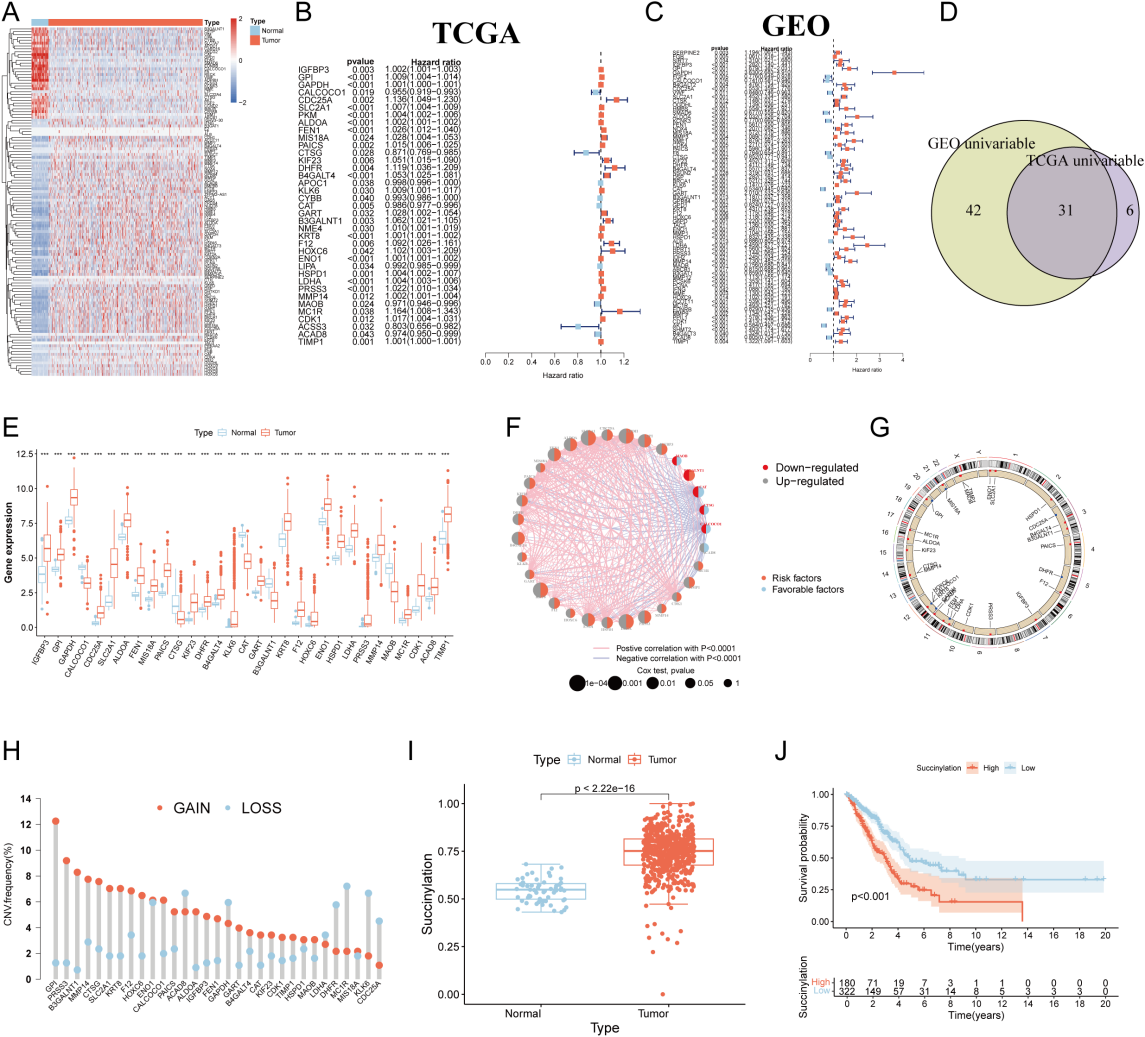
Identification of core succinylation-related genes in LUAD. **(A)** Heatmap displaying 91 upregulated (red) and 31 downregulated (blue) succinylation-related differentially expressed genes (DEGs) (FDR < 0.05 and |log_2_FC| > 1) in TCGA LUAD samples compared to normal tissues. Prognostic succinylation-related DEGs identified by univariable Cox regression analysis in TCGA **(B)** and merged GEO **(C)** cohorts. **(D)** Venn diagram illustrating the overlap of prognostic genes from TCGA and GEO cohorts, defining 31 core succinylation-related genes. **(E)** The differences of the 31 core succinylation-related genes expression between LUAD (red) and normal lung tissues (blue). **(F)** Correlation network of the 31 core succinylation-related genes. **(G)** Circular genome plot showing the chromosomal locations of the 31 core succinylation-related genes. **(H)** Bar plot demonstrating the prevalence of copy number variation (CNV) gain (red) and loss (blue) in the core genes in LUAD. **(I)** Succinylation scores (calculated by ssGSEA) are significantly elevated in LUAD compared to normal lung tissues (*p*< 2.2×10^-16^). **(J)** Kaplan-Meier survival curves indicating significantly poorer overall survival (OS) for LUAD patients with high succinylation scores (red) compared to those with low scores (blue) (log-rank test *p*< 0.001). “*” means that p <0.05; “**” means that p < 0.01; “***” means that p < 0.001; ns, no significance.

The flow chart of the study was showed in **Figure 1**. To systematically investigate the role of protein succinylation modification in LUAD, we initially curated a comprehensive set of 615 succinylation-related genes from the GeneCards database. Differential analysis between TCGA-LUAD samples and normal lung tissues revealed a substantial number of dysregulated succinylation-related genes, specifically 91 upregulated and 31 downregulated genes (**Figure 2A**). Subsequently, we assessed the prognostic value of these differentially expressed succinylation-related genes (screening criteria: FDR < 0.05 and |log_2_FC| > 1) through univariable Cox regression analysis in both the TCGA and a merged Gene Expression Omnibus (GEO) dataset. Thirty-seven prognostic genes in the TCGA cohort (**Figure 2B**) and 73 prognostic genes in the GEO cohort (**Figure 2C**) were identified. Thirty-one core succinylation-related genes were yielded by intersecting those prognostic genes (**Figure 2D**).

Expression disparities for these 31 core genes between LUAD and normal tissues were further visualized via a boxplot (**Figure 2E**), confirming that the majority were significantly upregulated in LUAD. A relevance and prognosis network unveiled complex relationships among these genes (**Figure 2F**). Genomic alteration analysis revealed the specific chromosomal locations of CNVs for these genes (**Figure 2G**) and indicated that their copy numbers were substantially altered in LUAD (**Figure 2H**).

Employing the ssGSEA algorithm, we quantified a succinylation score for each patient within the TCGA-LUAD cohort. This score was significantly elevated in LUAD patients compared to individuals with normal lung tissue (**Figure 2I**). Critically, survival analysis demonstrated that patients exhibiting high succinylation scores had a markedly worse OS than those with low succinylation scores (p < 0.001) (**Figure 2J**).

### 3.2 Distinct LUAD subtypes based on succinylation

**Figure 3:**
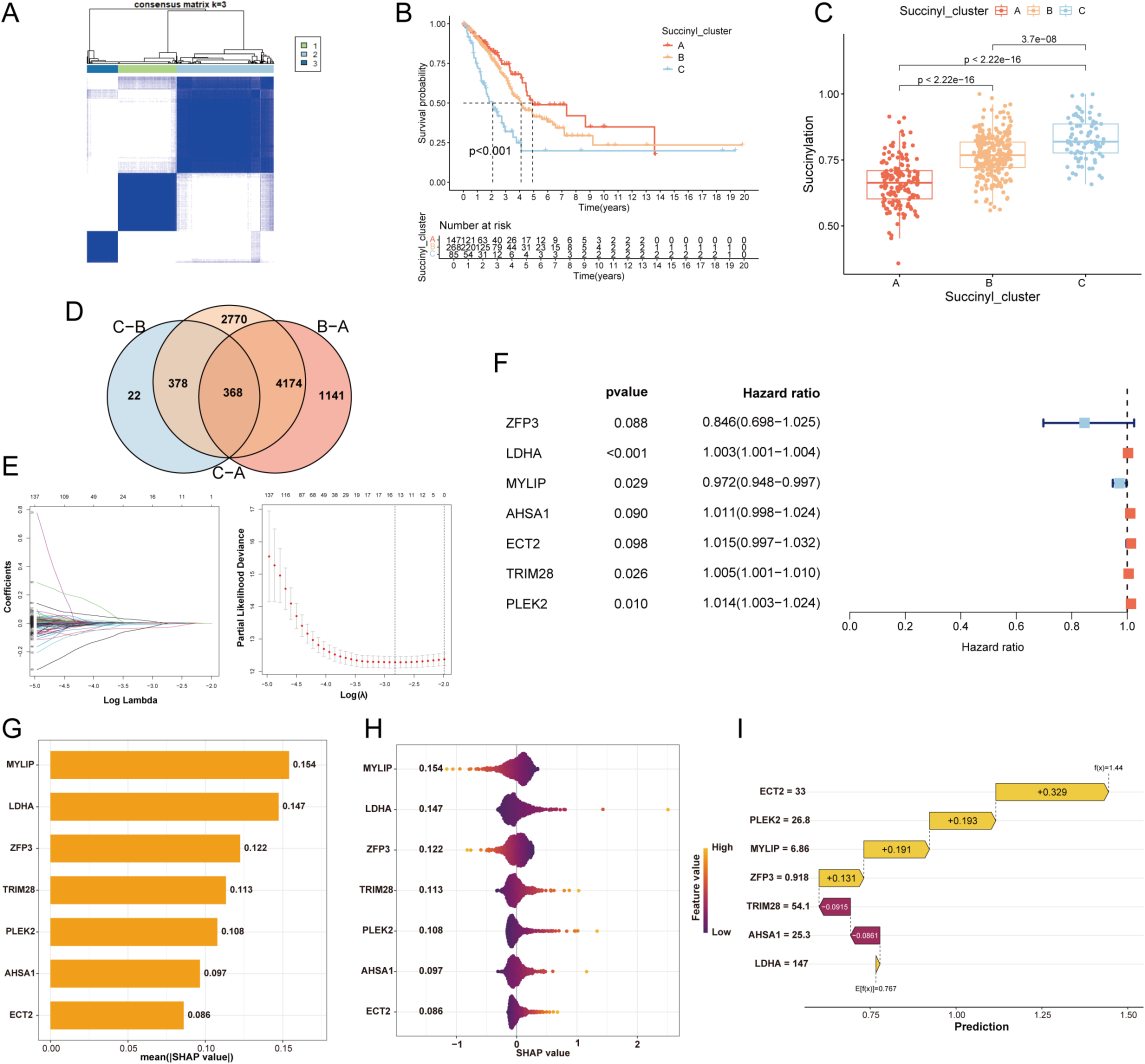
Development of a succinylation-related prognostic model for LUAD. **(A)** Consensus clustering matrix (k=3) of PCA demonstrating robust classification of LUAD samples into three subtypes **(A, B, C)** using the 31 core succinylation-related genes. **(B)** Kaplan-Meier OS curves showing significant prognostic differences among subtypes (log-rank test P< 0.001), with Subtype A associated with the most favorable outcome and Subtype C with the poorest. **(C)** Comparison of succinylation scores across the three subtypes. Subtype C exhibits the highest succinylation activity, while Subtype A shows the lowest (Wilcoxon rank-sum test all p< 0.001). **(D)** Venn diagram showing the overlap of DEGs (adjusted p< 0.01) among the three succinylation subtypes, identifying 368 common DEGs. **(E)** Feature selection using the least absolute shrinkage and selection operator (LASSO) Cox regression. **(F)** The final 7-gene prognostic signature identified by multivariable Cox regression analysis. **(G)** SHAP analysis displaying the mean absolute SHAP value for each feature, representing its relative importance in the model. **(H)** Violin plots of SHAP values for each gene in the model, illustrating the direction and magnitude of their impact on the risk score. **(I)** Waterfall plot for a representative patient (ID1), demonstrating the cumulative contribution of each model gene to the final calculated risk score.

To elucidate the heterogeneity of LUAD, we performed PCA based on the expression profiles of the 31 core succinylation-related genes. This analysis revealed three distinct molecular subtypes (Subtype A, B, and C) with clear separations (**Figure 3A**, **Supplementary Figure S1A**). Survival analysis indicated significant differences in OS among these subtypes, with Subtype A exhibiting the most favorable prognosis, followed by Subtype B, while Subtype C was associated with the poorest clinical outcomes (**Figure 3B**).

Consistent with the survival differences, evaluation of succinylation scores across subtypes showed that Subtype C possessed the highest succinylation activity, whereas Subtype A displayed the lowest (**Figure 3C**). A comprehensive heatmap further illustrated the distinct expression patterns of the 31 core succinylation-related genes and their correlations with key clinical features across the three subtypes (**Supplementary Figure S1B**). Analysis of tumor immune microenvironment via ssGSEA revealed distinct immune cell infiltration patterns among the subtypes. Subtype A was characterized by the highest infiltration levels of activated B cells, eosinophils, mast cells, and natural killer (NK) cells (**Supplementary Figure S1C**).

To investigate the underlying biological mechanisms driving the divergent prognoses, we conducted functional enrichment analyses. Subtype C, with the worst prognosis, showed significant enrichment in multiple oncogenic pathways, including DNA repair, reactive oxygen species pathway, mTORC1 signaling, and targets of E2F and MYC (**Supplementary Figure S1D**). KEGG pathway analysis further reinforced the involvement of cell cycle and various DNA repair processes in this aggressive subtype (**Supplementary Figure S1E**).

### 3.3 Development and validation of a prognostic model of succinylation

To develop a prognostic model of succinylation, we firstly identified 368 common DEGs among the three subtypes (p < 0.01; **Figure 3D**). GO analysis indicated that these genes were primarily involved in cell division-related activities such as chromosome segregation, organelle fission, and nuclear division. Their protein products were predominantly localized to chromosomal regions, spindles, and centrosomes, with molecular functions related to ATP hydrolysis activity, microtubule binding, and DNA helicase activity (**Supplementary Figure S1F**). KEGG pathway analysis demonstrated significant enrichment in critical oncogenic pathways including cell cycle, DNA replication, glycolysis, and HIF-1α signaling (**Supplementary Figure S1G**). Subsequent univariable Cox regression analysis revealed 60 genes significantly associated with prognosis (p < 0.05). To refine the feature set, LASSO regression was applied, reducing the candidate genes to 17 (**Figure 3E**). Finally, a prognostic model comprising 7 genes were developed by multivariable cox regression analysis. Among these, MYLIP expression was positively correlated with survival outcome, whereas elevated levels of LDHA, TRIM28, and PLEK2 were negatively associated with prognosis (**Figure 3F**). SHAP-based feature importance analysis indicated that MYLIP, LDHA, and ZFP3 were the top three contributors to the model’s predictive performance (**Figure 3G**). **Figure 3H** presents violin plots illustrating the distribution of SHAP values for each feature in the prognostic model, reflecting their relative contribution to prediction outcomes. Features with larger absolute SHAP values exert a greater influence on the model’s output, indicating higher importance in stratifying patient risk. Specifically, elevated expression of MYLIP and ZFP3 was associated with a reduction in the calculated risk score for LUAD patients, suggesting a protective prognostic role. Additionally, a waterfall plot delineates the cumulative contribution of each of the seven model genes in assigning a final risk classification for a representative patient (ID1), demonstrating the individual and collective impact of gene expression on the predicted outcome (**Figure 3I**).

Survival analysis demonstrated that patients in the high-risk group exhibited significantly worse OS and progression-free survival (PFS) in the TCGA cohort (**Supplementary Figures S2A, B**). These findings were consistently validated across seven independent GEO cohorts (GSE3141, GSE13213, GSE30219, GSE31210, GSE37745, GSE50081, and GSE72094) as well as in a merged GEO dataset, with all comparisons reaching statistical significance (**Supplementary Figures S2C–J**). To further evaluate the predictive accuracy of the prognostic model, ROC curves were generated. The model demonstrated robust discriminative ability for predicting 1-, 3-, and 5-year survival outcomes in both the TCGA and GEO cohorts. The area under the curve (AUC) values were as follows: TCGA-OS: 0.712, 0.699, 0.659; TCGA-PFS: 0.664, 0.689, 0.671; GSE3141: 0.693, 0.76, 0.839; GSE13213: 0.721, 0.69, 0.70; GSE30219: 0.746, 0.716, 0.761; GSE31210: 0.762, 0.732, 0.803; GSE37745: 0.622, 0.559, 0.629; GSE50081: 0.807, 0.773, 0.732; GSE72094: 0.666, 0.648, 0.669; GEO merge: 0.709, 0.668, 0.662) (**Supplementary Figures S2K–T**).

### 3.4 TME heterogeneity and immunotherapeutic benefits of LUAD patients in different risk groups

**Figure 4:**
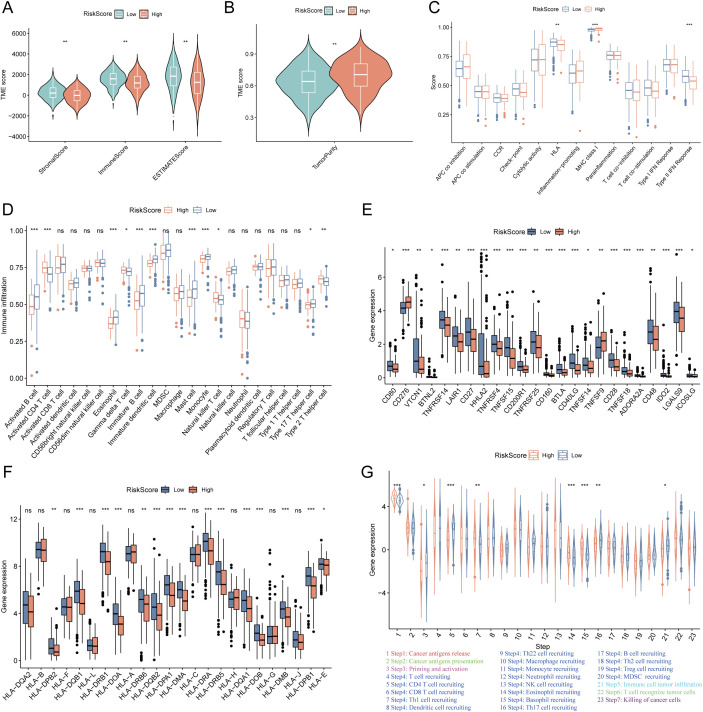
Tumor microenvironment (TME) heterogeneity and immune-related genes expression between low- and high-risk LUAD groups. **(A)** The differences of the stromal score, immune score, ESTIMATE score between high- and low-risk groups. The low-risk group exhibited significantly higher stromal, immune, and ESTIMATE scores (all *p* < 0.01). **(B)** The differences of the tumor purity between high- and low-risk groups. The low-risk group exhibited significantly lower tumor purity (*p* < 0.01). **(C)** The differences of immune-related functions between the high- and low-risk groups. **(D)** The differences of the infiltration levels of various immune cell types between the high- and low-risk groups. **(E)** The differences of immune checkpoints expression between the high- and low-risk groups. **(F)** The differences of the expression levels of HLA-related genes between the high- and low-risk groups. **(G)** The activity of key steps in the cancer-immunity cycle between the high- and low-risk groups. “*” means that p <0.05; “**” means that p < 0.01; “***” means that p < 0.001; ns, no significance.

To characterize the TME heterogeneity between high- and low-risk LUAD groups, we computed stromal, immune, ESTIMATE, and tumor purity scores. Results demonstrated that the low-risk group exhibited significantly higher stromal and immune scores, along with reduced tumor purity (all p< 0.01; **Figures 4A, B**), suggesting a more immunologically active TME. Analysis of immune-related functions revealed enhanced activity in HLA expression and type II IFN response in the low-risk group (**Figure 4C**). Furthermore, multiple immune cell types—including activated B cells, eosinophils, immature B cells, immature dendritic cells, mast cells, and monocytes—showed significantly elevated infiltration levels in the low-risk group (**Figure 4D**). Additionally, notable differences were observed in the expression of immunomodulatory molecules. Several inhibitory checkpoint genes (e.g., VTCN1, TNFRSF14, LAIR1, LGALS9) were upregulated in the low-risk group (**Figure 4E**), whereas PD-L1 protein expression was higher in the high-risk group (**Supplementary Figure S3A**). Concurrently, the low-risk group displayed elevated expression of HLA-related genes (**Figure 4F**), indicating a potentially enhanced antigen presentation capacity. The cancer–immunity cycle elucidates antitumor immune responses and offers an opportunity to understand the interactions between cancer and its immune system. Evaluation of the cancer–immunity cycle revealed that the low-risk group exhibited higher activity in key steps including CD4^+^ T cell recruitment, Th17 cell recruitment, and immune cell infiltration into tumors (**Figure 4G**). TIDE analysis further indicated a lower T cell exclusion score but higher T cell dysfunction score in the low-risk group (**Supplementary Figures S3B, C**). Consistent with these findings, the low-risk group was enriched for an immune-inflamed phenotype, whereas the high-risk group correlated with immune-desert or immune-excluded phenotypes. Additionally, immunophenoscore (IPS) was significantly higher in the low-risk group (**Supplementary Figures S3D, E**). Collectively, these results indicate that LUAD patients in the low-risk group possess a more immunoreactive TME with improved antigen presentation, immune cell infiltration, and enhanced activity across multiple steps of the cancer–immunity cycle, suggesting greater potential sensitivity to immunotherapy.

**Figure 5:**
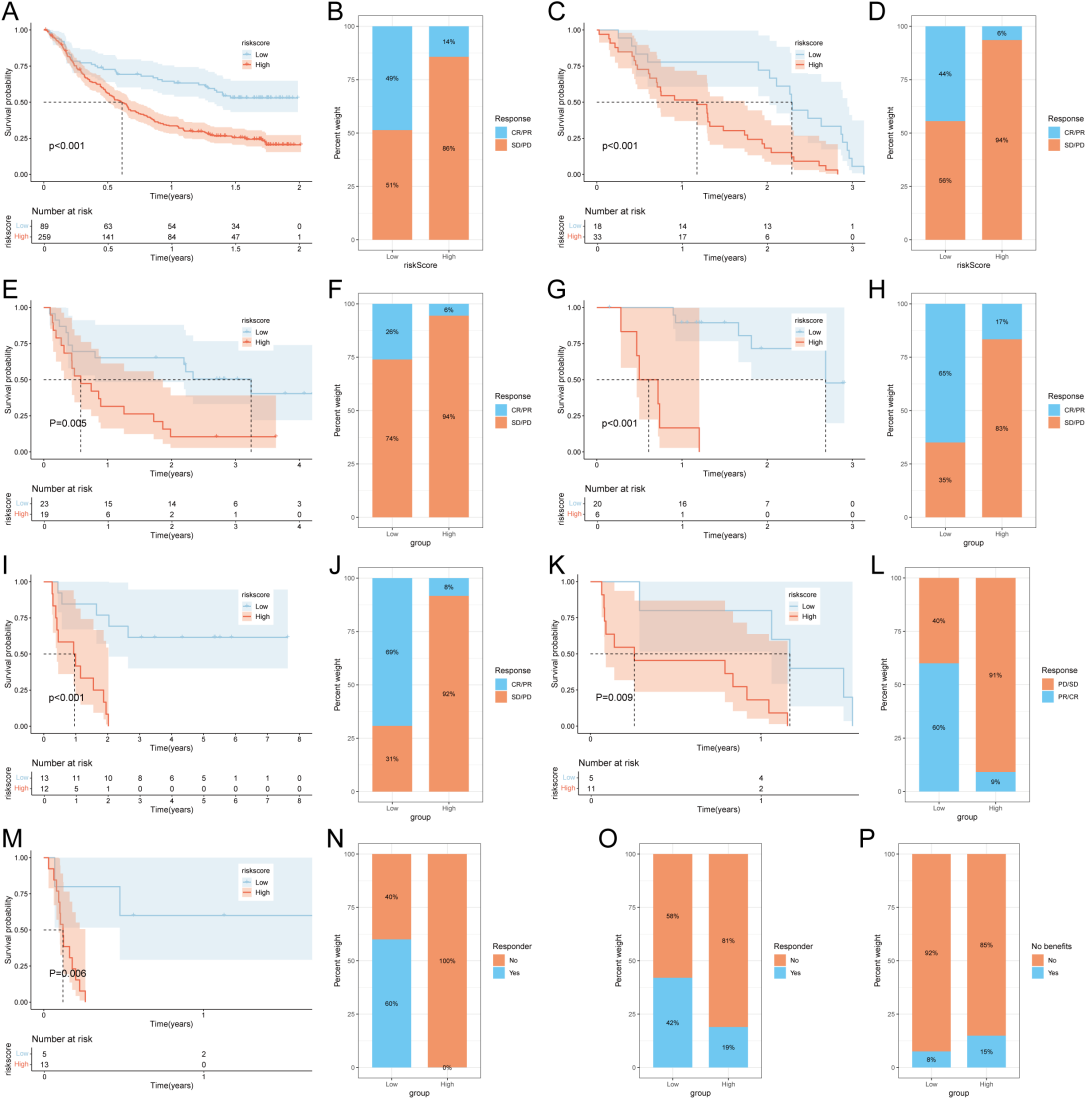
Prediction of immunotherapy response by succinylation-based model. Survival analysis **(A)** and response to anti-PD-L1 therapy **(B)** between the high- and low-risk groups in advanced urothelial cancer (IMvigor210 cohort). Survival analysis **(C)** and response to anti-CTLA4 and anti-PD1 therapy **(D)** between the high- and low-risk groups in melanoma (GSE91061). Survival analysis **(E)** and response to anti-CTLA4 therapy **(F)** between the high- and low-risk groups in metastatic melanoma. Survival analysis **(G)** and response to anti-PD-1 therapy **(H)** between the high- and low-risk groups in melanoma (GSE78220). Survival analysis **(I)** and response to adoptive T cell therapy **(J)** between the high- and low-risk groups in melanoma. Survival analysis **(K)** and response to anti-PD1 therapy **(L)** between the high- and low-risk groups in NSCLC (GSE126044). Survival analysis **(M)** and response to anti-PD-1/PD-L1 therapy **(N)** between the high- and low-risk groups in NSCLC (GSE135222). **(O)** Difference of responder between low- and high-risk group of LUAD in TCGA. **(P)** Difference of benefits between low- and high-risk group of LUAD in TCGA.

To further validate the prognostic value of the succinylation-related risk model in predicting immunotherapy response, we analyzed seven independent published cohorts encompassing diverse cancer types and immunotherapeutic regimens. These included: advanced urothelial cancer treated with atezolizumab (anti-PD-L1), melanoma treated with anti-CTLA4 and/or anti-PD-1 blockade, metastatic melanoma receiving anti-CTLA4 therapy, non-small cell lung cancer (NSCLC) treated with nivolumab or pembrolizumab (anti-PD-1), NSCLC under anti-PD-1/PD-L1 therapy, melanoma receiving ACT, and melanoma treated with anti-PD-1 monotherapy. Consistently across all validation cohorts, patients classified into the low-risk group demonstrated a significant survival advantage and higher objective response rates compared to those in the high-risk group (**Figures 5A–N**). Furthermore, using the TIDE algorithm applied to the TCGA-LUAD cohort, we predicted responses to anti-PD-1 and anti-CTLA4 therapies. The results indicated that patients in the low-risk group exhibited a higher likelihood of being classified as responders and deriving clinical benefit from immune checkpoint inhibitors (**Figures 5O, P**). These multi-cohort analyses robustly confirm that the succinylation-based risk stratification model effectively predicts immunotherapeutic outcomes, with the low-risk group associated with enhanced survival and improved response to various immunotherapy regimens.

### 3.5 TMB, tumor stemness and drug sensitivity analysis

**Figure 6:**
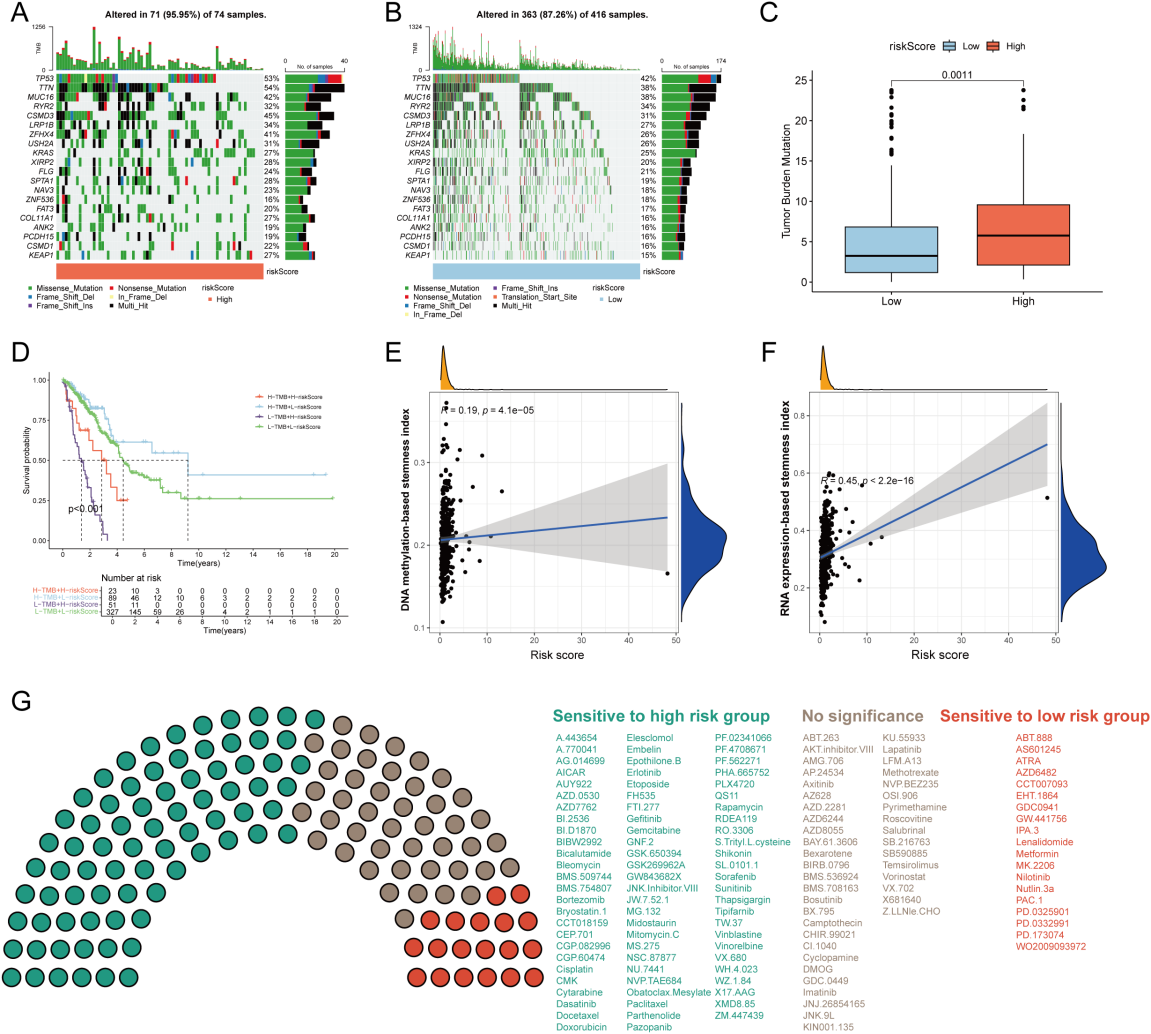
Tumor mutation burden (TMB) and drug sensitivity analysis. Visualization of the top 20 gene mutations in the high-risk group **(A)** and low-risk group **(B)**. **(C)** The differences of TMB between the high- and low-risk groups. **(C)** The Kaplan–Meier curves of OS in LUAD patients stratified by TMB and risk score. **(E)** Correlation of DNA methylation-based stemness index and risk score. **(F)** Correlation of RNA expression-based stemness index and risk score. **(G)** The sensitivity of 117 drugs between the high- and low-risk groups. *p* < 0.05 was considered as statistical significance.

To investigate somatic mutation patterns in LUAD with different risk score, we analyzed the distribution of mutations in high- and low-risk groups within the TCGA-LUAD cohort. The high-risk group exhibited a significantly higher overall mutation frequency (95.95%) compared to the low-risk group (87.26%). Key genes with notably elevated mutation rates in the high-risk group included TP53 (53% vs. 42%), TTN (54% vs. 38%), MUC16 (42% vs. 38%), CSMD3 (45% vs. 31%), and LRP1B (34% vs. 27%) (**Figures 6A, B**). Missense mutations and multi-hit events constituted the predominant mutation types in both groups. Consistent with the mutation burden, TMB was significantly higher in the high-risk group (p = 0.0011; **Figure 6C**). Kaplan–Meier survival analysis revealed that patients with low-risk scores and high TMB had the most favorable survival outcomes, whereas those with high-risk scores and low TMB exhibited the poorest prognosis (p < 0.001; **Figure 6D**). Furthermore, the risk score showed a significant positive correlation with tumor stemness indices derived from both DNA methylation (DNAss) and RNA expression (RNAss) data (**Figures 6E, F**). To evaluate the potential clinical utility of the risk model for guiding therapeutic strategies, we assessed the sensitivity of 137 chemotherapeutic and targeted agents across the two risk groups. Patients in the high-risk group demonstrated lower IC_50_ values (indicating higher sensitivity) to 77 drugs, whereas the low-risk group showed increased sensitivity to 19 agents (**Figure 6G**). These findings suggest distinct drug response patterns between the subgroups and may inform personalized treatment selection for LUAD patients.

### 3.6 Development and validation of a succinylation-based prognostic nomogram

To establish a clinically applicable succinylation-based nomogram, we evaluated the predictive value of the risk score along with key clinical variables—age, gender, pathological stage, and treatment type—using univariable and multivariable Cox regression analyses. Univariable analysis identified age (Hazard Ratio (HR) = 1.43, 95% Confidence Interval (CI): 1.056–1.936; p = 0.021), pathological stage (HR = 1.625, 95% CI: 1.414–1.869; p < 0.001), and risk score (HR = 1.121, 95% CI: 1.090–1.153; P< 0.001) as significantly associated with OS in LUAD (**Supplementary Figure S4A**). Following adjustment via multivariable analysis, age (HR = 1.435, 95% CI: 1.057–1.947; P = 0.02), pathological stage (HR = 1.579, 95% CI: 1.370–1.820; P< 0.001), and risk score (HR = 1.111, 95% CI: 1.079–1.144; P< 0.001) remained independent prognostic factors and were incorporated into a succinylation-based nomogram (**Supplementary Figure S4B**).

The resulting nomogram provided quantitative predictions of 1-, 3-, and 5-year OS probabilities for LUAD patients (**Supplementary Figure S4C**). Survival stratification based on the nomogram score effectively distinguished patient outcomes, with higher scores correlating with poorer prognosis (**Supplementary Figure S4D**). Calibration curves demonstrated excellent agreement between predicted and observed survival rates at 1, 3, and 5 years (**Supplementary Figure S4E**). ROC curve analysis revealed that the nomogram exhibited superior predictive accuracy compared to individual predictors—including risk score, age, gender, stage, and treatment type—with AUC values of 0.759, 0.749, and 0.747 for 1-, 3-, and 5-year survival, respectively (**Supplementary Figures S4F–H**). DCA further confirmed the enhanced clinical net benefit of the nomogram over single-factor models across a range of threshold probabilities (**Supplementary Figure S4I**).

### 3.7 Single-cell transcriptomic analysis of succinylation heterogeneity in LUAD

**Figure 7:**
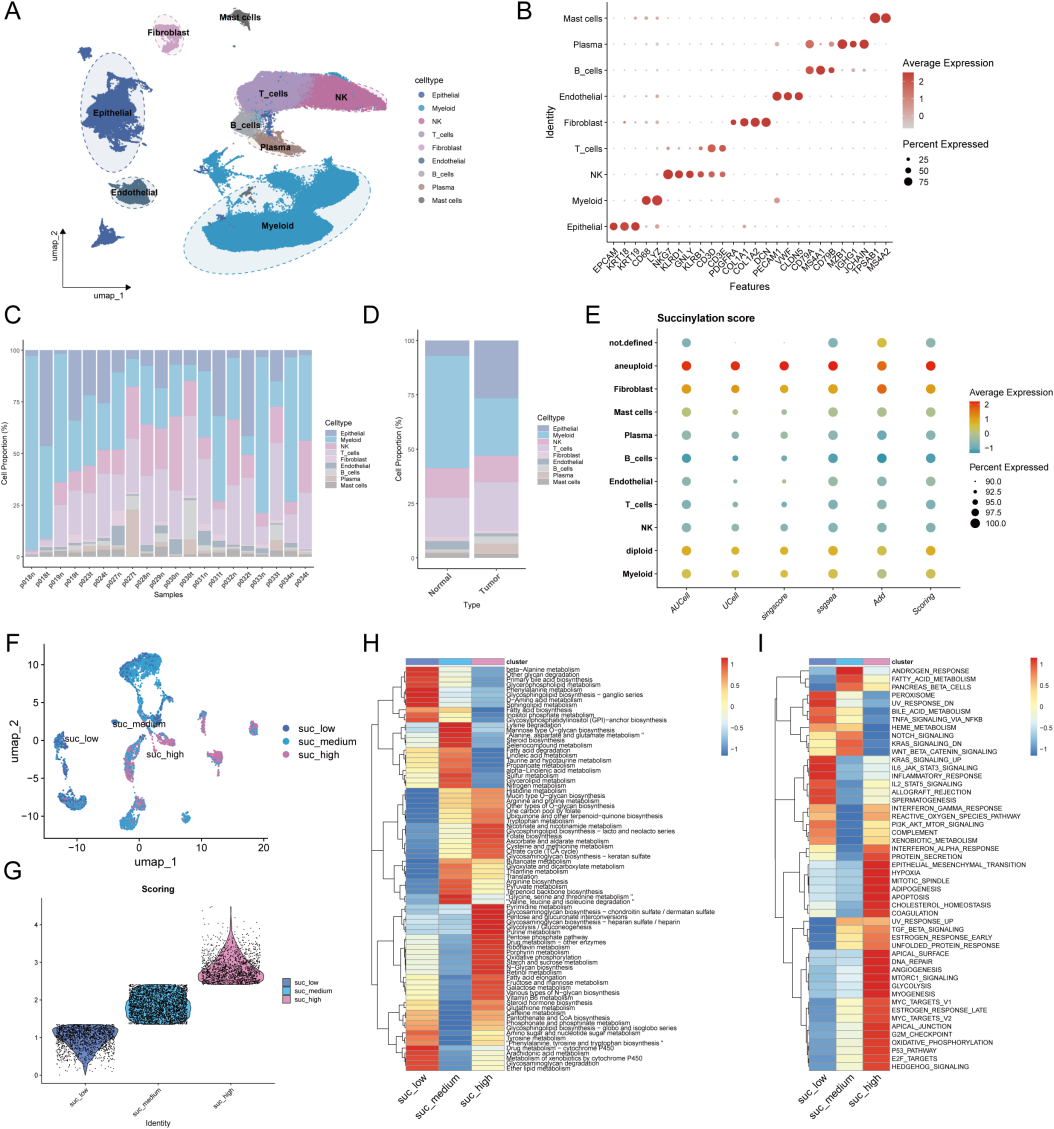
Single-cell transcriptomic profiles revealed the heterogeneity of succinylation in LUAD and its association with immunotherapy response. **(A)** UMAP plot of the nine major cell populations from 10 LUAD and 10 matched normal lung samples. **(B)** The average expression (color intensity) and percentage (dot size) of canonical marker genes for the nine major cell populations. The percentage of cellular composition across individual samples **(C)** and tissue types **(D)**. **(E)** The differences of the succinylation activity score across different cell types. Malignant epithelial cells exhibited the highest succinylation level. **(F)** The three subclusters of malignant epithelial cells based on succinylation score (low-succinylation, medium-succinylation, high-succinylation). **(G)** Violin plot shows the difference of succinylation score among three subclusters. The differences of 84 metabolic pathways **(H)** and Hallmark pathway analysis **(I)** among malignant epithelial cells with different succinylation levels.

To characterize succinylation heterogeneity at the single-cell level in LUAD, we analyzed a scRNA-seq dataset comprising 10 LUAD tissues and 10 matched normal lung samples. After quality control, a total of 120,435 high-quality cells were retained for downstream analysis. Cell types were annotated based on canonical markers into nine major populations: epithelial cells (EPCAM, KRT18, KRT19), myeloid cells (CD68, LYZ), NK cells (NKG7, KLRD1, GNLY, KLRB1), T cells (CD3D, CD3E), fibroblasts (PDGFRA, COL1A1, COL1A2, DCN), endothelial cells (PECAM1, VWF, CLDN5), B cells (CD79A, MS4A1, CD79B), plasma cells (MZB1, IGHG1, JCHAIN), and mast cells (TPSAB1, MS4A2) (**Figures 7A, B**). Sample-wise and tissue-type composition analysis revealed a predominant increase in epithelial cell proportion within LUAD samples (**Figures 7C, D**).

To distinguish malignant from normal epithelial cells, we applied the CopyKat algorithm, classifying epithelial cells into aneuploid (malignant, n = 6,284), diploid (normal lung epithelial, n = 12,443), and undefined (n = 2,541) subgroups. Succinylation activity was evaluated using a gene signature of 31 core succinylation-related genes, integrated from five computational methods (AUCell, UCell, SingScore, ssGSEA, and AddModuleScore). Malignant epithelial cells exhibited the highest succinylation scores, while immune populations—such as plasma cells, B cells, T cells, and NK cells—showed low activity (**Figure 7E**). Compared to normal lung tissue, LUAD samples displayed significantly elevated succinylation levels (**Supplementary Figure S5A**). According to succinylation scores, malignant epithelial cells were divided into low-, medium-, and high-succinylation subtypes (**Figures 7F, G**). Stratification of malignant cells into low-, medium-, and high-succinylation subtypes revealed distinct metabolic and functional states. High-succinylation cells were enriched in pathways including pyrimidine and purine metabolism, glycosaminoglycan biosynthesis, glycolysis, riboflavin/retinol/vitamin B6 metabolism, pentose phosphate pathway, and fatty acid metabolism (**Figure 7H**). Hallmark analysis further indicated elevated activity in oncogenic processes such as EMT, hypoxia response, TGF-β signaling, DNA repair, glycolysis, MYC targets, and E2F targets (**Figure 7I**). To further explore the function alteration among different succinylation subtypes, we performed the KEGG and GO enrichment analyses using upregulated genes in high-succinylation subtype and upregulated genes in low-succinylation subtype. The results of GO enrichment analysis showed that the upregulated genes in high-succinylation subtype were mainly related to ribosome biogenesis and mitochondrial gene expression while those in low-succinylation subtype were mainly related to vesicle and lysosome (**Supplementary Figures S6A, B**). The results of KEGG showed that the upregulated genes in high-succinylation subtype were mainly related to HIF-1α signaling and cancer while those in low-succinylation subtype were mainly related to endocytosis, lysosome and autophagy (**Supplementary Figures S6C, D**).

To assess the relevance of succinylation in immunotherapy response, we analyzed the NSCLC scRNA-seq dataset GSE207442, which includes samples from treatment-naïve and immunotherapy-treated patients. UMAP visualization confirmed cell-type annotations (**Supplementary Figure S5B**). Succinylation scores were significantly higher in treatment-naïve (TN) compared to post-immunotherapy samples, and non-major pathological response (NMPR) had higher succinylation scores than major pathological responders (MPR) (**Supplementary Figure S5C**). Consistent with prior findings, malignant cells exhibited the highest succinylation activity in this dataset (**Supplementary Figure S5D**).

### 3.8 Pseudotime trajectory and transcription factor analysis

**Figure 8:**
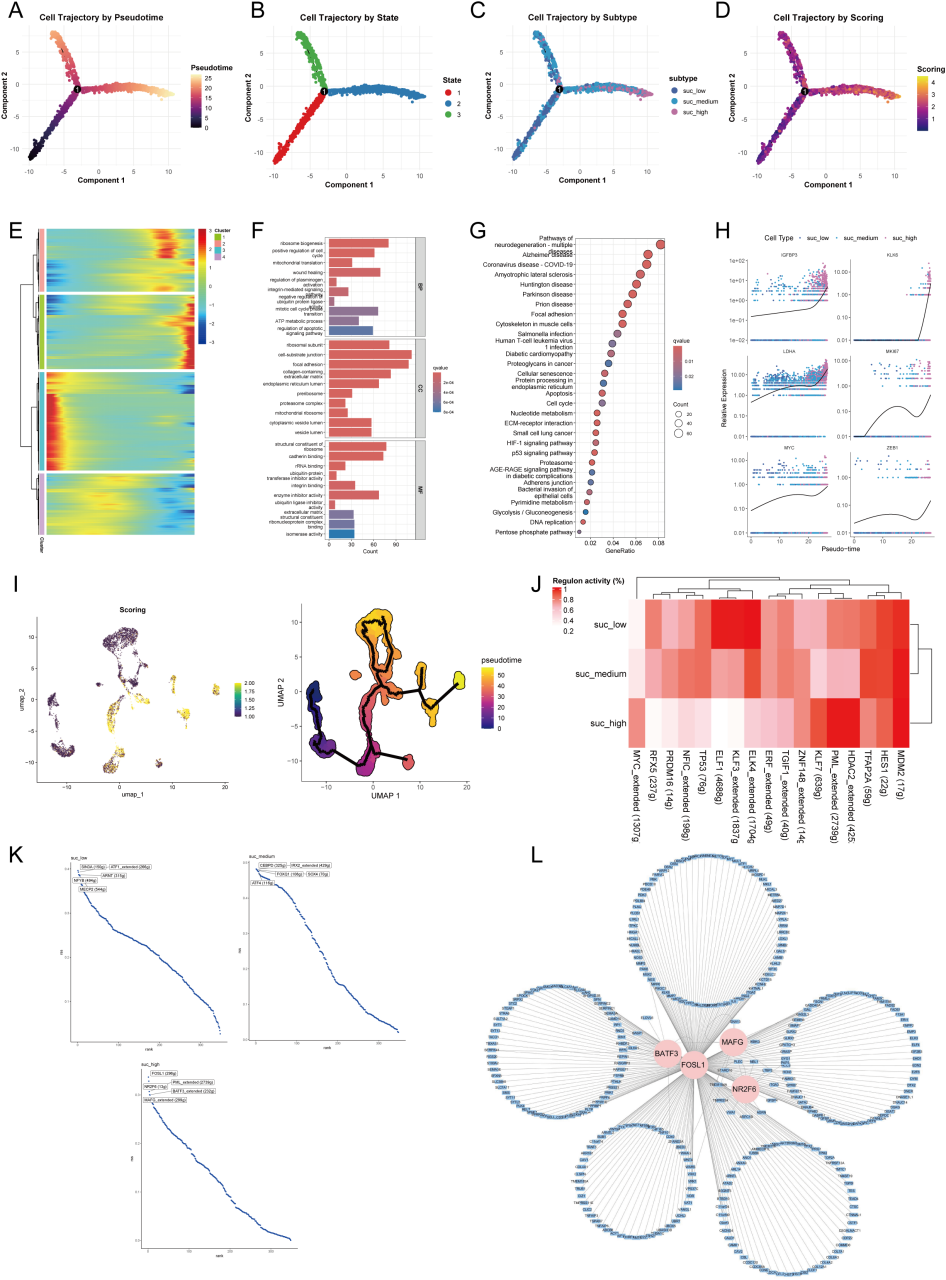
Pseudotime trajectory and transcriptional regulation of malignant epithelial cells across succinylation subclusters. Pseudotime analysis of malignant cells using Monocle. Cells are colored by inferred pseudotime **(A)**, cell state **(B)**, succinylation subclusters (low, medium, high) **(C)**, and succinylation score **(D)**. **(E)** Heatmap of gene expression dynamics along pseudotime, grouped into four distinct clusters. Cluster 1 shows highest expression at the trajectory endpoint. Functional enrichment analysis of Cluster 1 genes. Gene Ontology (GO) **(F)** terms and KEGG pathways **(G)** significantly associated with Cluster 1. **(H)** Expression patterns of selected succinylation-related genes (IGFBP3, KLK6, LDHA) and oncogenes (MKI67, MYC, ZEB1) along pseudotime. **(I)** Validation of the differentiation trajectory from low- to high- succinylation states using Monocle3. **(J)** Heatmap of transcription factor (TF) activity across succinylation subclusters by SCENIC analysis. **(K)** Top five TFs ranked by regulon activity in each succinylation subclusters. **(L)** Regulatory network of key TFs (BATF3, FOSL1, MAFG, NR2F6) and their target genes with high confidence by Cytoscape.

To delineate the dynamic transition of malignant cells with different succinylation levels, we reconstructed their pseudotime trajectory using Monocle. The developmental trajectory of malignant cells suggested three states (**Figures 8A, B**). The trajectory originated from malignant cells with low succinylation levels and progressively differentiated into populations with medium and high succinylation levels (**Figure 8C**). With prolonged pseudotime, the succinylation score in malignant cells became elevated (**Figure 8D**). A heatmap visualizing gene expression dynamics along the pseudotime axis revealed four distinct clusters. Cluster 1 was characterized by the highest gene expression levels at the terminal endpoint of the trajectory, suggesting its potential role in late-stage cellular processes and succinylation (**Figure 8E**). GO enrichment analysis indicated that the highly expressed genes in Cluster 1 were primarily associated with fundamental cellular processes, including transcription, translation, ATP metabolic process, pointing to heightened energy demands. Additionally, terms related to cell cycle progression, wound healing response, and integrin-mediated signaling pathway were significantly enriched, implying roles in proliferation and cellular communication (**Figure 8F**). Subsequent KEGG pathway analysis further demonstrated significant enrichment in pathways related to neurodegeneration diseases, diabetic cardiomyopathy, cellular senescence, nucleotide metabolism, ECM-receptor interaction, and the HIF-1 signaling pathway (**Figure 8G**). Notably, the expression of three core succinylation-related genes (IGFBP3, KLK6, LDHA) and three established oncogenic markers (MKI67, a proliferation index; MYC, a master transcription factor; ZEB1, an epithelial-mesenchymal transition regulator) was consistently upregulated along the pseudotime continuum (**Figure 8H**). This differentiation pattern, from low to high succinylation states, was corroborated by an independent analysis using Monocle3 (**Figure 8I**).

To identify key transcriptional regulators driving succinylation heterogeneity in malignant cells, we performed SCENIC analysis to infer TF activity. The analysis revealed that transcriptional activity of ELK4, KLF3, ELF1, and RFX5 was elevated in the low-succinylation group, whereas MDM2, HDAC2, PML, KLF7, and MYC exhibited higher activity in the high-succinylation group (**Figure 8J**). **Figure 8K** displays the top five TFs ranked by activity within each succinylation group. In the high-succinylation group, the top regulators were FOSL1, NR2F6, PML, BATF3, and MAFG. Using Cytoscape, we visualized the coregulatory network of BATF3, FOSL1, MAFG, and NR2F6 with their high-confidence target genes; PML was excluded from this network visualization due to a lack of high-confidence targets (**Figure 8L**).

### 3.9 Intercellular communication network

**Figure 9:**
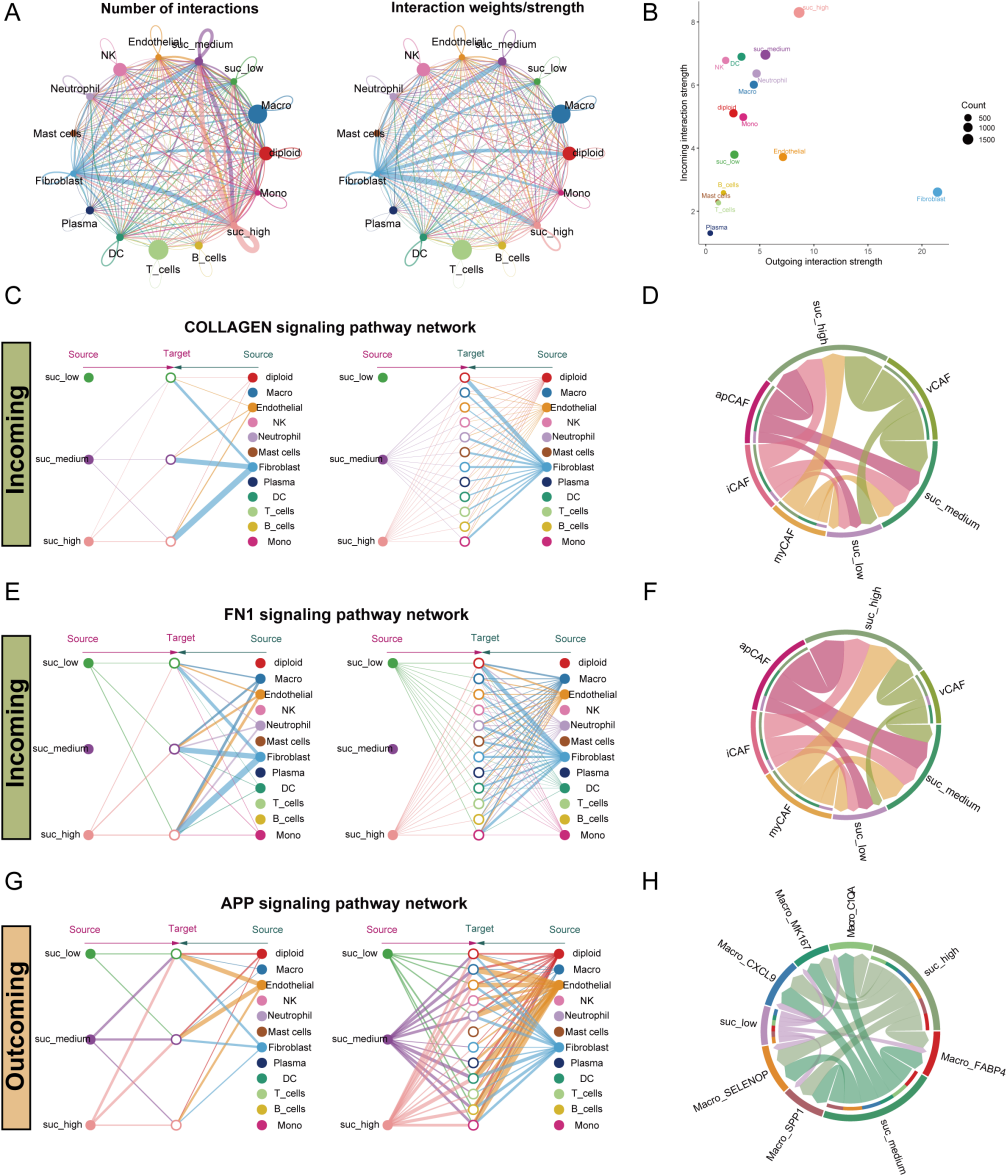
Intercellular communication pattern within the LUAD microenvironment across malignant epithelial cells with varying succinylation levels. **(A)** The interaction number and strength networks between malignant epithelial cells with varying succinylation levels and other cell types (stromal cells, immune cells). **(B)** The incoming and outgoing interaction strengths of each cell type. **(C)** Network diagrams of the collagen signaling pathways. Nodes represent cell types (source: signaling cells; target: recipient cells). Edge width corresponds to interaction strength. **(D)** Circular plots visualizing the subtype-specific engagement of cancer-associated fibroblasts (CAFs) (myCAF, iCAF, apCAF, vCAF) with malignant epithelial cells via the collagen signaling pathways. **(E)** Network diagrams of the FN1 signaling pathways. Nodes represent cell types (source: signaling cells; target: recipient cells). Edge width corresponds to interaction strength. **(F)** Circular plots visualizing the subtype-specific engagement of CAFs (myCAF, iCAF, apCAF, vCAF) with malignant cells via the FN1 signaling pathways. **(G)** Network diagrams of the APP signaling pathways. Nodes represent cell types (source: signaling cells; target: recipient cells). Edge width corresponds to interaction strength. **(H)** Circular plots visualizing the subtype-specific engagement of CAFs (myCAF, iCAF, apCAF, vCAF) with malignant cells via the APP signaling pathways. myCAF: myofibroblastic CAF; iCAF: inflammatory CAF; apCAF: antigen-presenting CAF; vCAF: vascular CAF.

To investigate cell-cell communication patterns within the LUAD microenvironment across malignant cells with varying succinylation levels, we constructed a comprehensive interaction network by Cellchat. This analysis revealed distinct communication profiles among the three succinylation clusters. Malignant cells exhibiting high succinylation scores demonstrated a greater number and stronger intensity of interactions with immune and stromal cells—particularly fibroblasts—compared to their low-succinylation counterparts (**Figure 9A**). Specifically, high-succinylation malignant cells displayed the strongest incoming interaction strength, whereas fibroblasts showed the most robust outgoing interaction strength (**Figure 9B**). Overall, high-succinylation malignant cells exhibited enhanced interaction strength in both incoming and outgoing signaling pathways (**Supplementary Figure S7A**). A detailed view of ligand-receptor pairs between malignant cells and other cell types is provided in **Supplementary Figures S8A, B**.

Notably, fibroblasts communicated differentially with malignant cells of different succinylation levels, especially through the collagen signaling pathway (**Figure 9C**). Further sub-clustering of fibroblasts into myCAF, iCAF, apCAF, and vCAF subtypes confirmed that all subtypes engaged in stronger interactions with high-succinylation malignant cells (**Figure 9D**). Expression profiles of key ligand-receptor pairs within the collagen pathway are visualized in **Supplementary Figure S7B**. A similar pattern was observed in the FN1 signaling pathway, where fibroblasts showed markedly stronger communication with high-succinylation malignant cells (**Figure 9E**). Among subtypes, iCAF, apCAF, and vCAF all exhibited enhanced interaction with high-succinylation malignant cells (**Figure 9F**), with corresponding ligand-receptor expression detailed in **Supplementary Figure S7C**.

In outgoing signaling, high-succinylation malignant cells preferentially communicated with other cells—such as macrophages and monocytes—via the APP signaling pathway (**Figures 9G, H**). These malignant cells also showed significantly stronger interactions across multiple macrophage subtypes and expressed higher levels of APP compared to low-succinylation cells (**Supplementary Figure S7D**).

### 3.10 Spatial landscape of succinylation of LUAD

**Figure 10:**
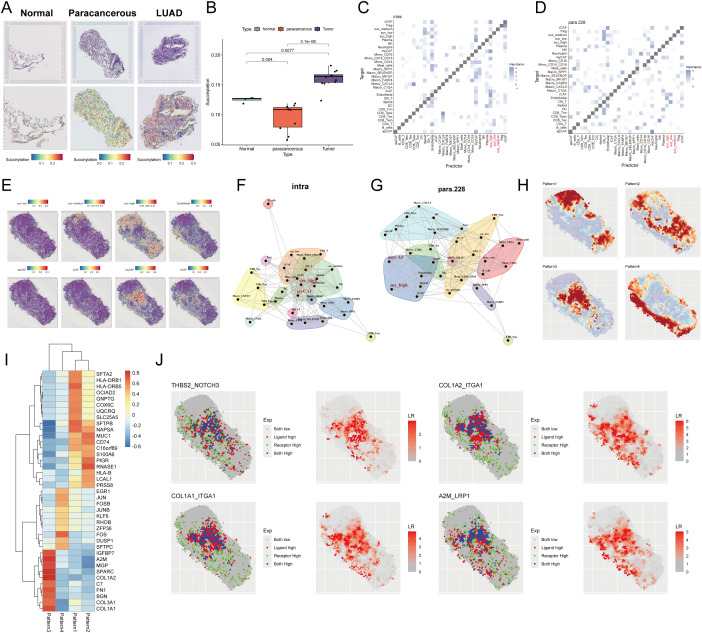
Spatial transcriptomic profiling reveals succinylation in LUAD. **(A)** Representative H&E-stained images (upper panel) and corresponding spatial distribution of succinylation scores (lower panel) for normal, paracancerous, and LUAD tissue sections. **(B)** Quantitative comparison of succinylation scores across normal, paracancerous, and LUAD tissue. MISTy framework analysis showing co-localization matrices for intracellular interactions (intrinsic view) **(C)** and microenvironment-influenced interactions (paraview) **(D)** in LUAD. **(E)** Spatial distribution map of succinylation scores across a representative tissue section in LUAD. Network diagrams from MISTy’s intrinsic view **(F)** and paraview **(G)**, illustrating spatial proximity between different cell types. **(H)** Spatially variable gene expression patterns identified by Spagen, revealing four distinct clusters across the tissue landscape. **(I)** Heatmap of the top 10 differentially expressed genes for each spatial pattern cluster. **(J)** Ligand-receptor interaction analysis highlighting significant pairs in the spatial landscape (including THBS2-NOTCH3, COL1A2-ITGA1, COL1A1-ITGA1, A2M-LRP1).

To investigate the spatial landscape of succinylation in LUAD, we performed spatial transcriptomic analysis on a dataset (E-MTAB-13530). Our findings demonstrated that LUAD tissue slides exhibited a significantly higher succinylation score compared to both normal and paracancerous tissue slides (**Figures 10A, B**). To resolve the cellular composition of the spatial transcriptomics data, we employed RCTD using a pre-annotated scRNA-seq dataset as a reference. To quantitatively assess the impact of cellular spatial organization on intercellular communication, we utilized the MISTy framework and constructed an intrinsic view to capture intracellular relationships and a paraview to model the influence of the immediate cellular microenvironment. Analysis of the intrinsic view revealed that malignant cells with elevated succinylation scores showed increased co-localization with various CAF subtypes, including myCAF, apCAF, iCAF, and vCAF (**Figure 10C**). This positive correlation between succinylation levels and spatial proximity to CAFs was further validated by the paraview analysis (**Figure 10D**). Notably, among the CAF subtypes, high-succinylation malignant cells demonstrated the closest spatial proximity to myCAFs (**Figures 10E–G**).

Subsequently, we utilized Spagen to identify spatially variable genes, which revealed four distinct spatial expression patterns across the tissue sections (**Figure 10H**). A heatmap displayed the top 10 spatially variable genes associated with each pattern (**Figure 10I**). Finally, ligand-receptor interaction analysis highlighted several significant interactions, prominently featuring the pairs THBS2-NOTCH3, COL1A2-ITGA1, COL1A1-ITGA1, and A2M-LRP1 (**Figure 10J**), suggesting active cross-talk between specific cell populations within the spatially resolved succinylation-rich niches of the LUAD TME.

### 3.11 Identification of potential targets related to succinylation in LUAD

**Figure 11:**
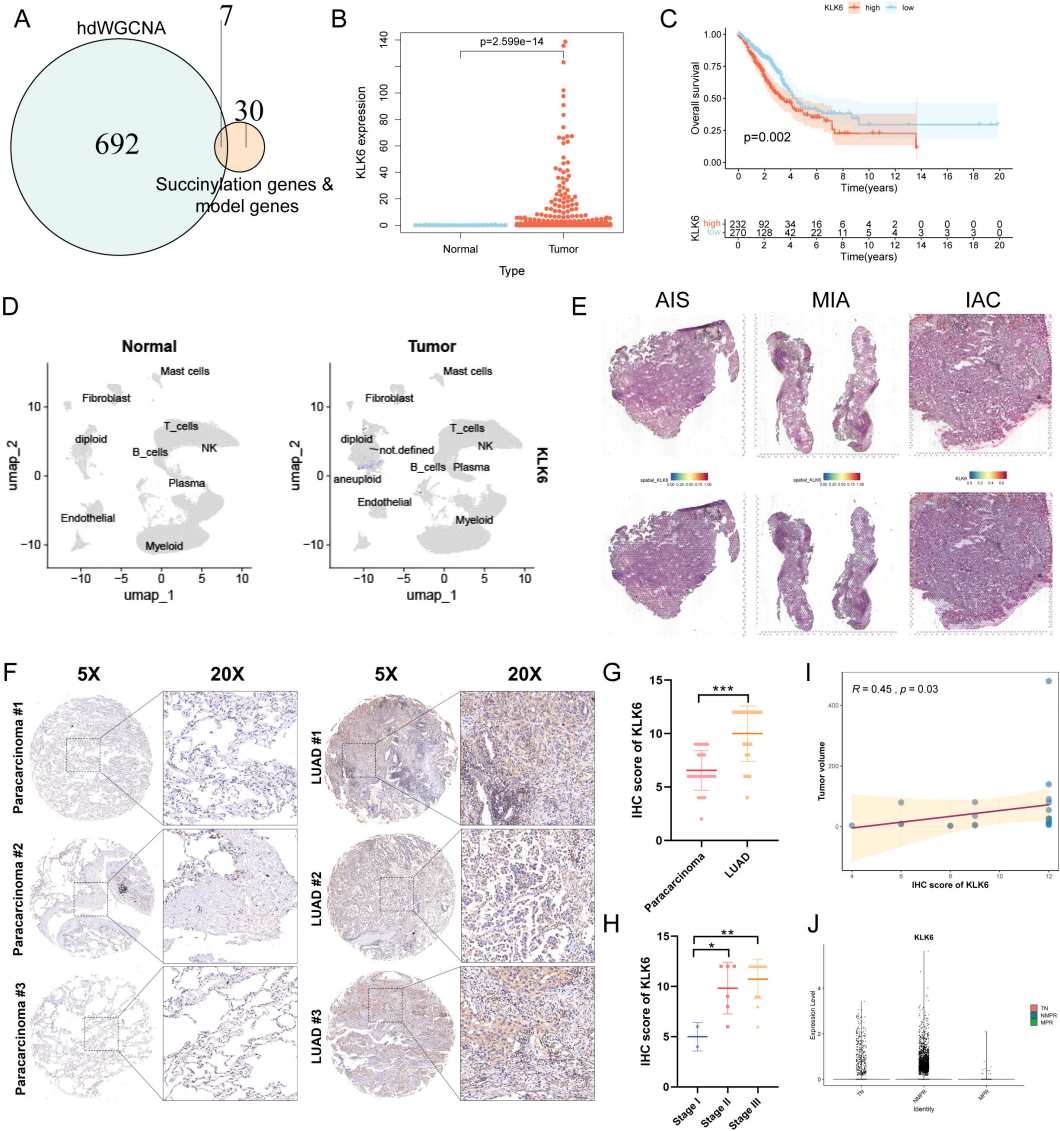
KLK6 is highly expressed in LUAD and correlates with Poor Prognosis. **(A)** Venn diagram illustrating the intersection between succinylation-related module genes (699 genes), core succinylation genes (31 genes), and model genes (7 genes), resulting in seven key candidate genes. **(B)** KLK6 mRNA expression is significantly upregulated in LUAD tissues compared to normal lung tissues. **(C)** Kaplan–Meier survival analysis of LUAD patients stratified by KLK6 expression level. High KLK6 expression is associated with significantly worse OS (log-rank test, *p* = 0.002). **(D)** UMAP plot shows KLK6 is significantly elevated in LUAD compared to normal lung tissues. **(E)** Spatial landscape revealed a gradual increase in KLK6 expression that paralleled the advancement of malignant progression—from pre-invasive *in situ* adenocarcinoma (AIS) to minimally invasive adenocarcinoma (MIA) and finally to invasive adenocarcinoma (IAC). **(F)** Representative IHC images of LUAD and paracarcinoma. **(G)** The differences of IHC score of KLK6 between LUAD and paracarcinomas. **(H)** The differences of IHC score of KLK6 across different pathological stage. **(I)** The correlation of IHC score of KLK6 and tumor size by Spearman’s rank correlation. *R* = 0.45, p = 0.03. **(J)** The differences of KLK6 expression among the treatment-naïve (TN) group, non-major pathological response (NMPR) group, and major pathological response (MPR) group. “*” means that p <0.05; “**” means that p < 0.01; “***” means that p < 0.001.

To systematically identify potential therapeutic targets associated with succinylation in LUAD, we performed hdWGCNA to identify functional modules linked to succinylation. The soft power threshold was set to 8 to achieve a scale-free topology network, and the resulting dendrogram illustrated the hierarchical clustering of genes (**Supplementary Figure S9A**). A total of 19 distinct co-expression modules were identified and eight modules—cyan, brown, blue, lightcyan, grey60, purple, lightyellow, and green—were selected as succinylation-related modules due to their significant correlation with succinylation scores (**Supplementary Figure S9B**). This selection yielded 699 succinylation-related genes. Subsequent intersection of these genes with 31 core succinylation genes and established model genes identified seven key genes: SLC2A1, KLK6, ENO1, HSPD1, LDHA, PRSS3, and ACAD8 (**Figure 11A**). The expression of the 7 key genes was displayed (**Supplementary Figure S9C**), with KLK6 exhibiting specific expression in malignant epithelial cells. Furthermore, a positive correlation between the expression levels of these genes and succinylation scores in malignant cells was revealed (**Supplementary Figure S9D**), suggesting their potential roles in succinylation-driven oncogenesis. The specific enrichment of KLK6 in malignant cells underscores its potential as a target for precision therapy, as kallikrein family members have been implicated in tumor progression and immune modulation. As a result, we focused KLK6 in the following study.

### 3.12 KLK6 Is highly expressed in luad and correlates with poor prognosis

Differential expression analysis revealed that KLK6 mRNA levels were significantly elevated in LUAD tissues compared to normal lung tissues (**Figure 11B**, **Supplementary Figure S9E**). LUAD patients with high KLK6 expression exhibited significantly poorer OS than those with low expression (**Figure 11C**). The results of scRNA-seq analysis demonstrated that KLK6 was predominantly expressed in malignant epithelial cells and was markedly upregulated in LUAD samples compared to normal lung tissues (**Supplementary Figure S9F**, **Figure 11D**). Tracking KLK6 expression across the histopathological continuum of lung adenocarcinoma development—from pre-invasive *in situ* adenocarcinoma (AIS) to minimally invasive adenocarcinoma (MIA) and finally to invasive adenocarcinoma (IAC) by spatial transcriptomics—revealed a gradual increase in KLK6 expression that paralleled the advancement of malignant progression (**Figure 11E**), underscoring its potential role in driving tumor invasion and aggressiveness. To further validate the protein expression of KLK6, we stained a IHC microarray of 30 LUAD and 30 matched paracancinoma. Due to peeling during the dyeing process, 23 LUAD and 30 paracarcinoma were retained. The protein levels of KLK6 in LUAD were significantly elevated compared to the paracarcinomas (**Figure 11F**). LUAD samples had higher IHC score of KLK6 than the paracarcinomas (*p* < 0.001) (**Figure 11G**). The IHC score of KLK6 was gradually increased paralleling the advancement of LUAD pathological stage (**Figure 11H**). Moreover, The IHC score of KLK6 was positively related to tumor size (*R* = 0.45, *p* = 0.03) (**Figure 11I**). Furthermore, we observed that the NMPR group had significantly higher KLK6 expression levels than both the TN and MPR groups (**Figure 11J**). This suggests that KLK6 may contribute to therapy resistance.

### 3.13 KLK6 expression remodeled the intercellular communication network in LUAD

**Figure 12:**
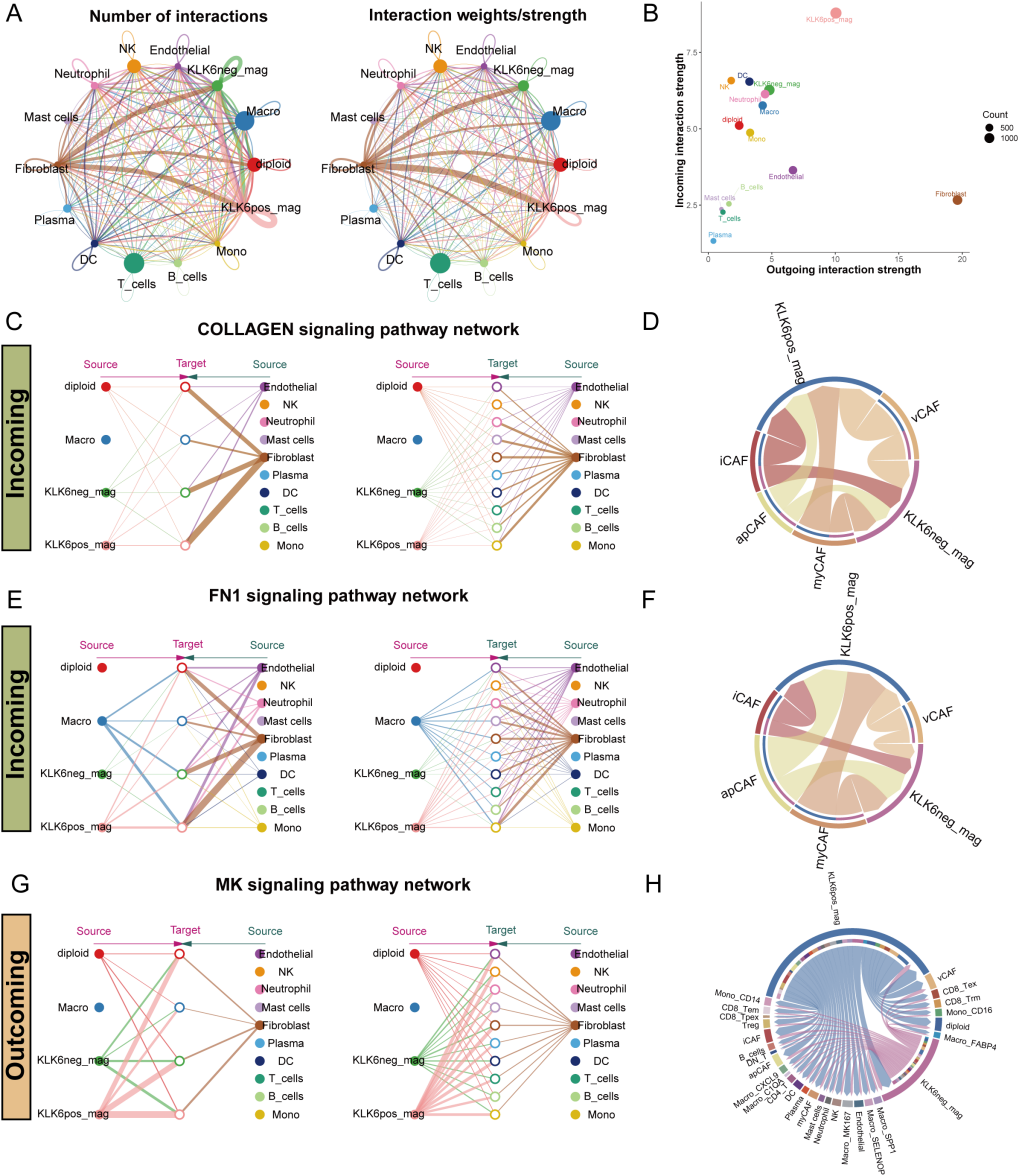
KLK6 expression remodeled the intercellular communication network in LUAD. **(A)** The interaction number and strength networks between malignant epithelial cells with different KLK6 expression and other cell types (stromal cells, immune cells). **(B)** The incoming and outgoing interaction strengths of each cell type. **(C)** Network diagrams of the collagen signaling pathways. Nodes represent cell types (source: signaling cells; target: recipient cells). Edge width corresponds to interaction strength. **(D)** Circular plots visualizing the subtype-specific engagement of CAFs (myCAF, iCAF, apCAF, vCAF) with malignant epithelial cells via the collagen signaling pathways. **(E)** Network diagrams of the FN1 signaling pathways. Nodes represent cell types (source: signaling cells; target: recipient cells). Edge width corresponds to interaction strength. **(F)** Circular plots visualizing the subtype-specific engagement of CAFs (myCAF, iCAF, apCAF, vCAF) with malignant epithelial cells via the FN1 signaling pathways. **(G)** Network diagrams of the MK signaling pathways. Nodes represent cell types (source: signaling cells; target: recipient cells). Edge width corresponds to interaction strength. **(H)** Circular plots visualizing the subtype-specific engagement of CAFs (myCAF, iCAF, apCAF, vCAF) with malignant epithelial cells via the MK signaling pathways.

To systematically evaluate the impact of KLK6 expression on the TME, we classified malignant cells into KLK6-positive (expression > 0) and KLK6-negative (expression = 0) populations and interrogated their intercellular communication networks using CellChat. Our analysis revealed that KLK6-positive malignant cells engage in a significantly more complex and robust interaction network with immune and stromal cells—particularly fibroblasts—compared to KLK6-negative cells (**Figure 12A**). Specifically, KLK6-positive malignant cells exhibited the strongest incoming interaction strength, indicating their heightened responsiveness to signals from the microenvironment, while fibroblasts displayed the most potent outgoing interaction strength, highlighting their role as active signal initiators (**Figure 12B**). A detailed visualization of ligand-receptor pairs between malignant cells and other cell types is provided in **Supplementary Figures S10A, B**.

Fibroblasts demonstrated markedly differential communication with KLK6-positive versus KLK6-negative malignant cells, with the collagen signaling pathway emerging as a predominant axis of interaction (**Figure 12C**). Further stratification of fibroblasts into canonical subtypes—myCAF, iCAF, apCAF, and vCAF—confirmed that all subtypes engaged in significantly stronger interactions with KLK6-positive malignant cells (**Figure 12D**). Expression profiles of key ligand-receptor pairs within the collagen pathway were visualized in **Supplementary Figure S11A**. A parallel enhancement in communication strength was observed in the FN1 signaling pathway, where fibroblasts again showed preferential signaling toward KLK6-positive malignant cells (**Figure 12E**). Among fibroblast subtypes, iCAF, apCAF, and vCAF uniformly exhibited intensified interactions with KLK6-positive malignant cells (**Figure 12F**), with corresponding ligand-receptor expression detailed in **Supplementary Figure S11B**.

In outgoing signaling, KLK6-positive malignant cells preferentially communicated with other cells via the midkine (MK) signaling pathway (**Figures 12G, H**). These cells demonstrated significantly higher expression of MK signaling signatures compared to KLK6-negative malignant cells (**Supplementary Figure S11C**), suggesting an active role in modulating the microenvironment. These findings position KLK6 not merely as a biomarker but as a functional orchestrator of tumor-stromal crosstalk, potentially driving LUAD progression through multidimensional network remodeling.

### 3.14 KLK6 expression reshapes the spatial landscape of luad by Modulating tumor-stromal interactions

**Figure 13:**
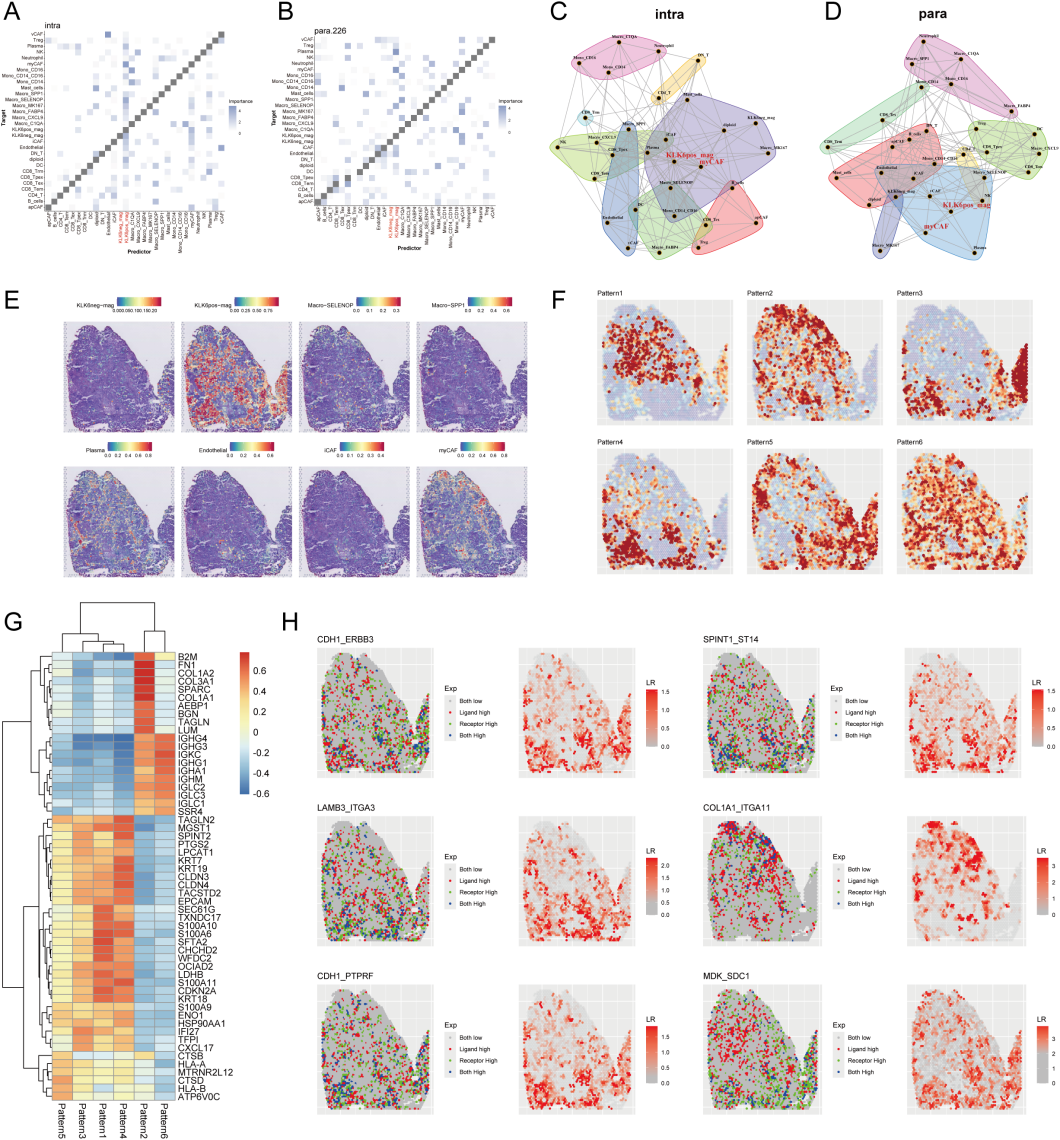
KLK6 expression reshapes the spatial landscape of LUAD by modulating tumor-stromal interactions. MISTy framework analysis showing colocalization matrices for intracellular interactions (intrinsic view) **(A)** and microenvironment-influenced interactions (paraview) **(B)** in LUAD. Network diagrams from MISTy’s intrinsic view **(C)** and paraview **(D)**, illustrating spatial proximity between different cell types. **(E)** Spatial distribution map of malignant cells with distinct KLK6 expression, immune cells, endothelial cells and CAFs across a representative tissue section in LUAD. **(F)** Spatially variable gene expression patterns identified by Spagen, revealing six distinct clusters across the tissue landscape. **(G)** Heatmap of the top 10 differentially expressed genes for each spatial pattern cluster. **(H)** Ligand-receptor interaction analysis highlighting significant pairs (including CDH1-ERBB3, LAMB3-ITGA3, CDH1-PTPRF, SPINT1-ST14, COL1A1-ITGA11, and MDK-SDC1) in spatial landscape of LUAD.

To systematically investigate the impact of KLK6 on the spatial architecture of LUAD, we employed the MISTy framework to analyze intracellular relationships (intrinsic view) and microenvironmental influences (paraview). Our analysis revealed that KLK6-positive malignant cells exhibited significantly enhanced spatial co-localization with multiple stromal and immune cell populations compared to KLK6-negative cells. In the intrinsic view, KLK6-positive cells showed increased proximity to cancer-associated fibroblasts (myCAF, iCAF), plasma cells, SELENOP+ macrophages, SPP1+ macrophages, MK167+ macrophages, and endothelial cells (**Figure 13A**). The paraview analysis further demonstrated strengthened interactions with regulatory T cells (Tregs), SELENOP+ macrophages, myCAFs, iCAFs, and endothelial cells (**Figure 13B**), indicating that KLK6 expression promotes a multicellular immunosuppressive niche. Spatial interaction networks for both views were visualized in **Figures 13C, D**. The distribution of KLK6-positive malignant cells, KLK6-negative malignant cells, myCAFs, SELENOP+ macrophages, SPP1+ macrophages, plasma cells, endothelial cells, iCAFs, and myCAFs in LUAD slides was showed in the **Figure 13E**. To resolve region-specific gene expression patterns, we performed spatially variable gene analysis using Spagen, which identified six distinct spatial expression patterns (**Figure 13F**). A heatmap of the top 10 spatially variable genes per pattern highlighted transcriptional gradients associated with zones of immune infiltration, stromal activation, and tumor proliferation (**Figure 13G**). Ligand-receptor interaction analysis further identified key signaling axes preferentially active in KLK6-enriched regions, including CDH1-ERBB3, LAMB3-ITGA3, CDH1-PTPRF, SPINT1-ST14, COL1A1-ITGA11, and MDK-SDC1 (**Figure 13H**). These interactions suggest that KLK6-positive cells engage in autocrine and paracrine signaling that reinforces fibroblast differentiation, macrophage polarization, and T cell exhaustion—processes critical for metastatic progression and therapy resistance.

### 3.15 KLK6 promotes succinylation, proliferation, migration, and invasion of LUAD cells *in vitro*

**Figure 14:**
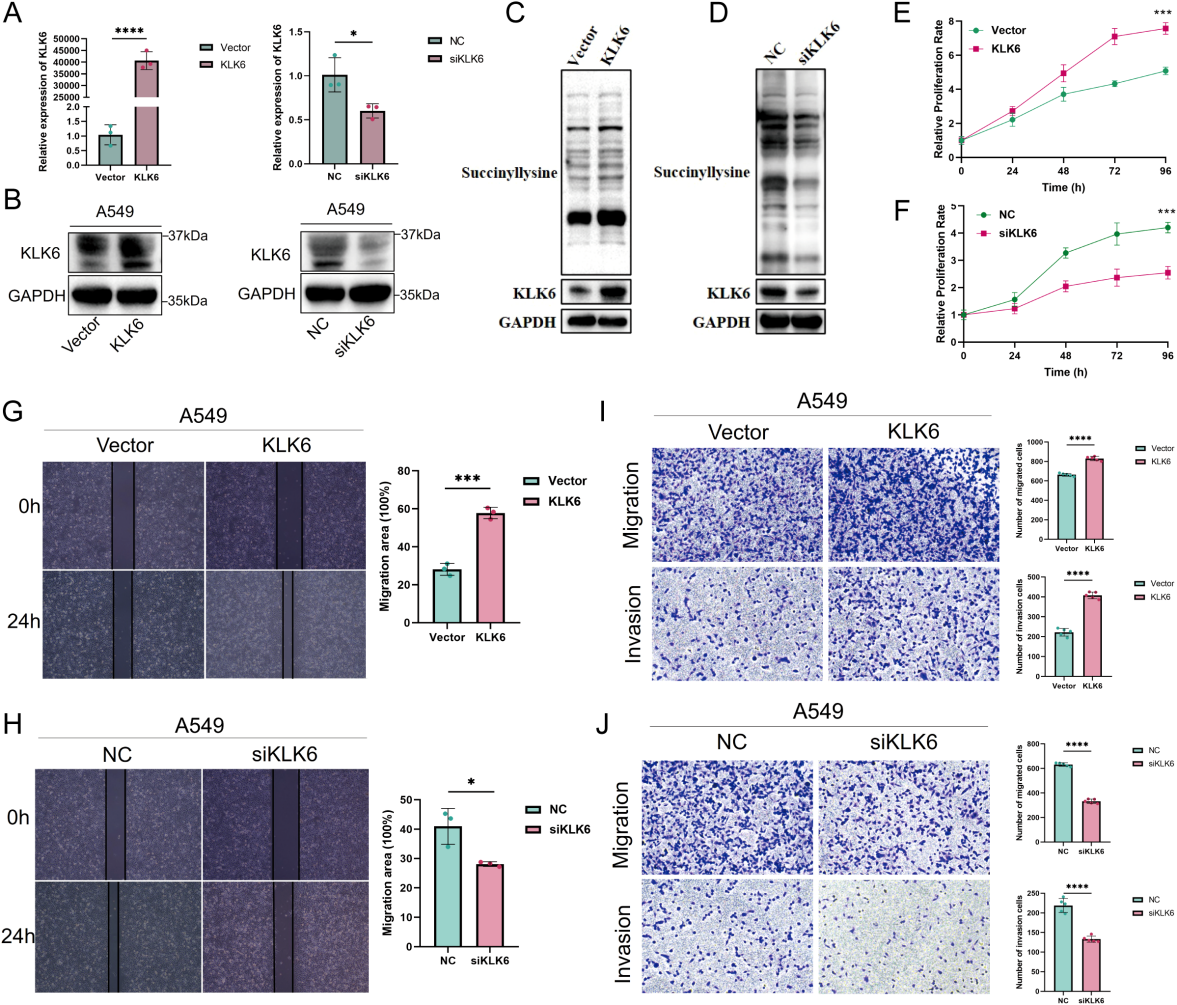
KLK6 promotes succinylation, proliferation, migration, and invasion of LUAD cells *in vitro.* KLK6 expression was modulated in A549 cells through overexpression and knockdown strategies. **(A)** qRT-PCR analysis showing mRNA levels of KLK6. **(B)** Western blot analysis confirming KLK6 protein expression. Vector: empty vector control; NC: negative control. **(C)** Western blot analysis confirming KLK6 overexpression enhanced levels of protein succinylation in A549 cells. **(D)** Western blot analysis confirming KLK6 silencing reduced levels of protein succinylation in A549 cells. **(E, F)** Cell proliferation was assessed by CCK-8 assay at indicated time points. KLK6 overexpression enhanced proliferation, while KLK6 silencing suppressed growth compared to controls. **(G, H)** Wound healing assays evaluating A549 cell migration when KLK6 overexpression and knockdown. Left: Representative images; right: quantitative analysis of wound closure at 0 h and 24 h. **(I, J)** Transwell migration and Matrigel invasion assays. Left: Representative images; right: quantification of migrated and invaded rate. Data are presented as mean ± SD from three independent experiments. “*” means that p <0.05; “**” means that p < 0.01; “***” means that p < 0.001.

To elucidate the pathological role of KLK6 in LUAD, we first established KLK6 overexpression and knockdown systems by using siRNA and plasmid in A549 cells, respectively. As shown in **Figures 14A, B**, the systems were successfully established with high overexpression and silencing efficiency. We found that overexpression of KLK6 significantly enhanced levels of protein succinylation whereas knockdown of KLK6 markedly reduced levels of protein succinylation in A549 cells (**Figures 14C, D**). Using the CCK-8 assay, we found that overexpression of KLK6 significantly enhanced the proliferation of A549 cells, whereas knockdown of KLK6 markedly suppressed proliferation (*p* < 0.05) (**Figures 14E, F**). Wound healing assays demonstrated that KLK6 overexpression accelerated wound closure, while KLK6 knockdown impaired the migratory capacity of A549 cells (**Figures 14G, H**; *p* < 0.05). Consistent with these findings, Transwell assays revealed that KLK6 overexpression significantly potentiated both migration and invasion of A549 cells, whereas KLK6 depletion impaired these invasive properties (**Figures 14I, J**). These results collectively establish that KLK6 functionally drives LUAD cell proliferation, migration, and invasion.

### 3.16 KLK6 promotes fibroblast-to-myofibroblast differentiation and may inhibit infiltration of CD8+ T cells

**Figure 15:**
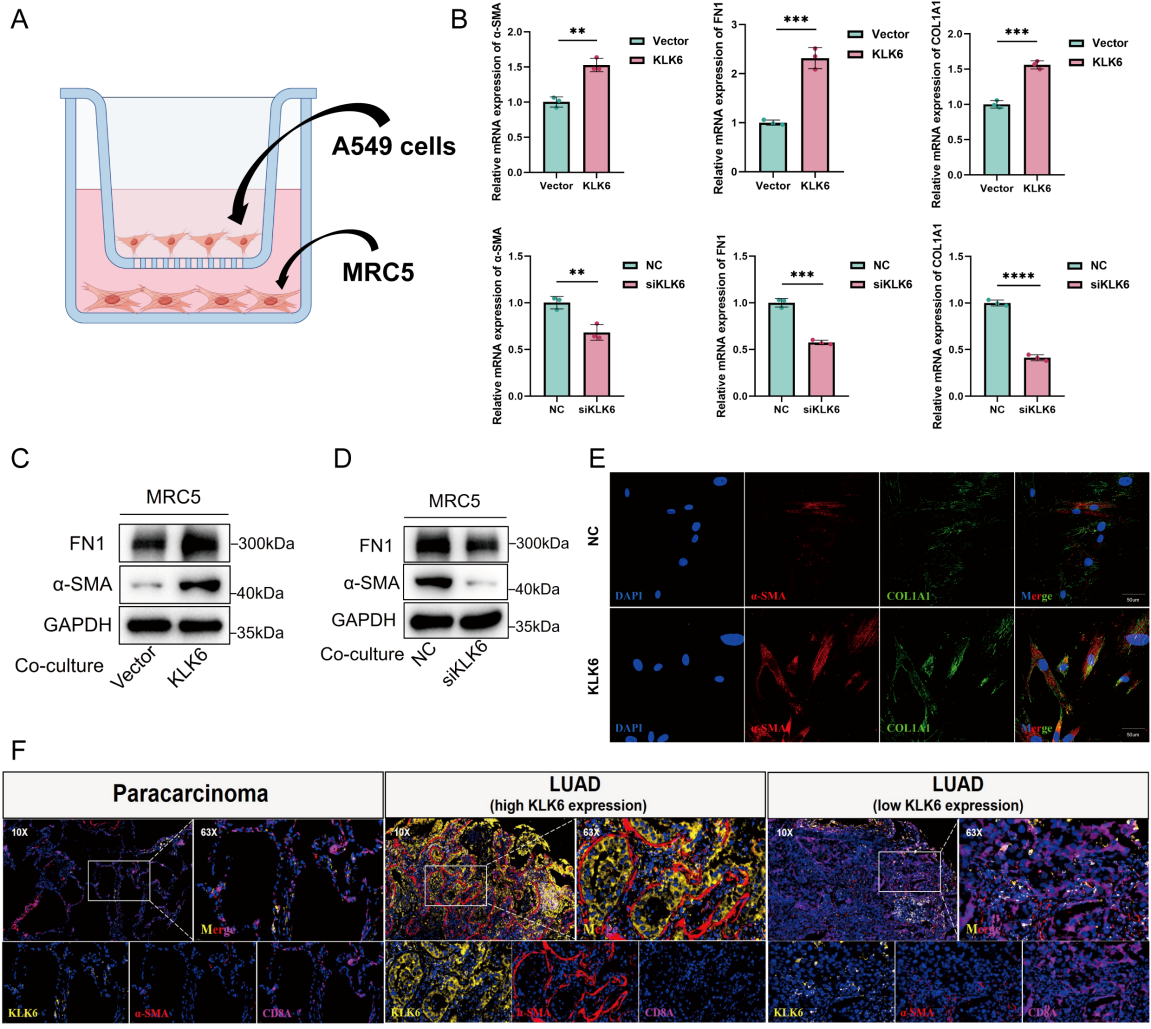
KLK6 promotes fibroblast-to-myofibroblast differentiation and modulates CD8+ T cell infiltration in LUAD. **(A)** Schematic diagram of the Transwell co-culture system using A549 cells (upper chamber) and MRC-5 cells (lower chamber). **(B)** RT-qPCR analysis of fibrotic markers (ACTA2/α-SMA, FN1, COL1A1) in MRC-5 cells co-cultured with KLK6-modulated A549 cells. KLK6 overexpression upregulates, while KLK6 knockdown downregulates, mRNA expression of these markers. **(C, D)** Western blot analysis confirms corresponding changes in α-SMA and FN1 protein expression in MRC-5 cells under KLK6-altered co-culture conditions. **(E)** Immunofluorescence staining showing α-SMA (red) and COL1A1 (green) expression in MRC-5 cells. Nuclei were counterstained with DAPI (blue). Scale bar: 50 μm. **(F)** Multiplex immunofluorescence of human LUAD tissues and paired adjacent normal tissues. High KLK6 expression correlates with increased α-SMA+ myofibroblasts and reduced CD8A+ T cell infiltration. Data are presented as mean ± SD from three independent experiments. “*” means that p <0.05; “**” means that p < 0.01; “***” means that p < 0.001.

To investigate the potential role of KLK6-positive malignant epithelial cells in promoting fibroblast-to-myofibroblast differentiation, we established a co-culture system using A549 cells and MRC-5 cells (**Figure 15A**). Our results demonstrated that KLK6 overexpression in A549 cells significantly upregulated the mRNA expression of fibrotic markers, including ACTA2 (α-SMA), FN1, and COL1A1, in co-cultured MRC-5 cells. Conversely, KLK6 knockdown in A549 cells led to a marked reduction in ACTA2 FN1, and COL1A1 mRNA levels in MRC-5 cells (**Figure 15B**). Consistent with these findings, western blot analysis confirmed that KLK6 overexpression enhanced the protein expression of α-SMA and FN1 in MRC-5 cells (**Figure 15C**), whereas KLK6 suppression attenuated their expression (**Figure 15D**). Immunofluorescence staining further revealed increased expression of α-SMA and COL1A1 in MRC-5 cells co-cultured with KLK6-overexpressing A549 cells compared to controls (**Figure 15E**).

To validate these observations *in vivo*, we performed multiplex immunofluorescence staining on human LUAD tissues and paired adjacent normal tissues. The analysis indicated that KLK6 expression was significantly higher in LUAD tissues than in paracancerous tissues. Furthermore, regions with high KLK6 expression exhibited elevated levels of the myofibroblast marker α-SMA and reduced infiltration of CD8+ T cells, as indicated by decreased CD8A expression (**Figure 15F**). These data collectively suggest that KLK6 in LUAD cells promotes fibroblast-to-myofibroblast transition and may contribute to an immunosuppressive microenvironment by limiting CD8+ T cell infiltration.

## 4 Discussion

LUAD remains a leading cause of cancer-related mortality worldwide (1). While immune checkpoint inhibitors (ICIs) have improved outcomes for a subset of patients, the majority exhibit limited responses due to primary or acquired resistance (4). This highlights the critical need to identify novel mechanisms underlying immunotherapy resistance and to develop robust biomarkers for patient stratification. Recent evidence implicates post-translational modifications, particularly succinylation, as key regulators of TME and anti-tumor immunity (14). Succinylation dynamically alters protein function by introducing a negatively charged succinyl group to lysine residues, modulating metabolic pathways, epigenetic landscapes, and immune cell interactions (32–34). In LUAD, aberrant succinylation promotes metabolic reprogramming and suppresses antitumor immunity, suggesting its potential as a therapeutic target to overcome immunotherapy resistance (35). However, a comprehensive understanding of the succinylation landscape in LUAD and its impact on patient prognosis and therapy response has been lacking.

In this study, we systematically characterized the role of protein succinylation in LUAD. We identified 31 core succinylation-related genes with significant prognostic value, revealing that elevated succinylation activity is a hallmark of aggressive disease and is associated with an immunosuppressive TME. We further established a novel succinylation-based prognostic model that effectively stratifies patients into distinct risk groups. This model demonstrated consistent predictive power for OS and PFS across multiple independent cohorts from TCGA and GEO. More importantly, it proved to be a robust predictor of response to immunotherapy. Patients in the low-risk group exhibited a more immunoreactive TME characterized by enhanced antigen presentation, greater infiltration of various immune cells, and higher activity in key steps of the cancer-immunity cycle, which correlated with improved survival benefits from ICIs across several validation cohorts. This suggests that our succinylation signature captures critical biological features of the TME that influence therapeutic efficacy. The similar mechanisms have been reported in other malignancies. For instance, in melanoma, CPT1A-mediated succinylation of PD-L1 accelerates its degradation via the lysosomal pathway, enhancing T-cell cytotoxicity and response to anti-PD-1 therapy (13). In glioblastoma, KAT2A/α-KGDH complex-driven histone H3 succinylation at K79 activates oncogenic transcription (36), while in hepatocellular carcinoma, OXCT1-succinylated LACTB reinforces mitochondrial metabolism to support tumor progression (10). These findings underscore the conserved yet context-dependent role of succinylation across cancers.

Our study identified KLK6 as a pivotal molecule associated with protein succinylation and the progression of LUAD. KLK6 expression was markedly elevated in malignant epithelial cells and increased with advancing tumor stage. High KLK6 levels correlated with poor prognosis and resistance to immunotherapy. KLK6 positively regulated levels of protein succinylation in A549 cells. Currently, there is no evidence that KLK6 directly regulates protein succinylation. Therefore, we hypothesize that KLK6 may modulate this modification through the following potential mechanisms: Hypothesis 1: As a secreted protease, KLK6 may cleave and activate cell surface protease-activated receptors (e.g., PAR1), thereby activating EGFR and MAPK signaling pathways and upregulating c-Myc expression. c-Myc directly promotes mitochondrial biogenesis, function, and increases the intracellular pool of succinyl-CoA, the essential substrate for protein succinylation (37). Hypothesis 2: KLK6 might hydrolyze a regulatory or binding protein of SIRT5, the primary cellular desuccinylase, which inhibits SIRT5 activity or its transport. Hypothesis 3: KLK6 could directly cleave succinyltransferases or desuccinylases themselves, thereby altering their activity, stability, or subcellular localization, and indirectly influencing the overall succinylation. Overexpressing KLK6 promoted LUAD cells proliferation, migration, and invasion *in vitro*. Moreover, KLK6 expression reshaped the intercellular communication network within the TME. Specifically, KLK6-positive malignant cells exhibited intensified interactions with CAFs, especially myCAFs, primarily through collagen and FN1-mediated signaling pathways. By a co-culture system using A549 and MRC-5 cells and multiple IF staining, we also found KLK6 in LUAD cells promoted fibroblast-to-myofibroblast transition and might prevent infiltration of CD8+ T cells. MyCAFs are characterized by high expression of α-SMA, fibroblast activation protein (FAP), and collagen I, and play a critical role in ECM remodeling, tumor invasion, and resistance (38). ECM secreted by MyCAFs provides structural support and adhesion sites for tumor cells, facilitating tumor architecture maintenance and tumor cell survival (38). Furthermore, increased matrix stiffness activates mechanosensitive signaling pathways in tumor cells, such as Hippo-YAP, Wnt/β-catenin, and PI3K-Akt, via integrin receptors, thereby driving proliferation, invasion of tumor cells (39–41). Additionally, the dense ECM acts as a physical barrier that limits drug penetration into the tumor core, thereby diminishing therapeutic efficacy and promoting chemoresistance. It also mechanically obstructs the infiltration of cytotoxic T cells, enabling immune evasion by tumor cells (42). Interestingly, myCAFs also have been reported to reprogram the metabolic landscape in TME. To accommodate the augmented glutamine demand of tumor cells, myCAFs actively upregulate anabolic pathways and secrete glutamine into TME, thereby sustaining its supply (43). Furthermore, myCAFs can inhibit the oxidative phosphorylation process in tumor cells through exosome-mediated mechanisms, which subsequently promotes glutamine-dependent reductive carboxylation (44, 45). The spatial co-localization of KLK6-positive cells with SELENOP+ and SPP1+ macrophages is also particularly noteworthy, as these subsets are known to exhibit immunosuppressive properties and promote tumor angiogenesis (46, 47). Similarly, interactions with Tregs indicate that KLK6 may contribute to an immune-excluded phenotype, potentially explaining the poor response to immune checkpoint inhibitors observed in KLK6-high LUAD patients. The prominence of MDK-SDC1 signaling aligns with KLK6’s role in enhancing MK-mediated pathways, which can further amplify inflammatory and pro-tumorigenic signals in the microenvironment.

Consequently, a self-sustaining vicious cycle is established: KLK6-positive tumor cells drive fibroblast-to-myofibroblast differentiation, which in turn secrete excessive ECM components. This aberrant ECM remodeling may foster a pro-tumorigenic microenvironment that facilitates disease progression, induces local immunosuppression, and promotes therapy resistance.

Despite these findings, our study has several limitations. First, while we constructed a prognostic model and validated it in public datasets, prospective validation in large, multi-center clinical cohorts is necessary to confirm its clinical utility before any potential clinical application due to the shared biases raised by public cohorts. Second, the specific molecular mechanisms linking KLK6 to the regulation of protein succinylation remain to be fully elucidated. Whether KLK6 directly influences succinylation or is a downstream effector requires further experimental investigation. Third, validating the effects of KLK6 on LUAD in *in vivo* models would strengthen our conclusions. Finally, the molecular mechanism of KLK6 positive LUAD cells promoting fibroblast-to-myofibroblast transition remains to be fully elucidated.

## 5 Conclusion

In conclusion, our work delineates a comprehensive landscape of enhanced succinylation modification in LUAD, establishes a succinylation-related model predicting prognosis and immunotherapy response, and identifies KLK6 as a key player in promoting LUAD progression and high KLK6 expression is associated with an immunosuppressive TME characterized by reduced CD8+ T cell infiltration, potentially mediated through the promotion of CAF differentiation and ECM remodeling. These findings provide a foundation for developing succinylation-based stratification strategies and highlight KLK6 as a potential therapeutic target for overcoming immunotherapy resistance in LUAD.

## Data Availability

The datasets presented in this study can be found in online repositories. The names of the repository/repositories and accession number(s) can be found in the article/**Supplementary Material**.

## Supplementary material

Supplementary Figure 1 Molecular subtyping of lung adenocarcinoma based on 31 core succinylation-related genes reveals distinct functional, and immune characteristics. **(A)** Three-dimensional PCA plot validating clear separation of the three identified molecular subtypes. **(B)** Heatmap integrating the expression patterns of the 31 core succinylation-related genes with key clinical-pathological features across the three subtypes. **(C)** Immune cell infiltration landscape assessed by ssGSEA among three subtypes. **(D)** Hallmark gene set analysis for Subtype C compared to subtype A. **(E)** KEGG pathway analysis for Subtype C compared to subtype A. **(F)** Gene Ontology (GO) analysis of common differentially expressed genes across all subtypes. **(G)** KEGG pathway analysis of common differentially expressed genes across all subtypes. PCA, principal component analysis; KEGG, Kyoto Encyclopedia of Genes and Genomes; GO, Gene Ontology; ssGSEA, single-sample gene set enrichment analysis. “*” means that p <0.05; “**” means that p < 0.01; “***” means that p < 0.001; ns, no significance.

Supplementary Figure 2 Predictive performance of the succinylation-related model. Kaplan-Meier survival curves comparing OS and progression-free survival (PFS) between high-risk and low-risk groups across different cohorts. Patients in the high-risk group shows significantly poorer outcomes in the **(A)** TCGA-OS, **(B)** TCGA-PFS, **(C)** GSE3141, **(D)** GSE13213, **(E)** GSE30219, **(F)** GSE31210, **(G)** GSE37745, **(H)** GSE50081, **(I)** GSE72094, and **(J)** merged GEO datasets (Log-rank test, all p< 0.001). Time-dependent receiver operating characteristic (ROC) curves assessing the predictive accuracy of the model for 1-, 3-, and 5-year survival. The area under the curve (AUC) values for each time point are indicated in the respective cohorts: **(K)** TCGA-OS, **(L)** TCGA-PFS, **(M)** GSE3141, **(N)** GSE13213, **(O)** GSE30219, **(P)** GSE31210, **(Q)** GSE37745, **(R)** GSE50081, **(S)** GSE72094, and **(T)** GEO merge.

Supplementary Figure 3 TIDE score and IPS difference between low- and high-risk LUAD groups. **(A)** PD-L1 protein expression was elevated in the high-risk group than the low-risk group. LUAD patients in the high-risk group had higher T cell exclusion **(B)** but lower T cell dysfunction scores **(C)** from the TIDE analysis. LUAD patients in the low-risk group had higher IPS with CTLA4- and PD-1- **(D)**, CTLA4+ and PD-1- **(E)**.

Supplementary Figure 4 Development and evaluation of a prognostic nomogram. Univariable **(A)** and multivariable **(B)** Cox regression analyses of risk score and clinical features. **(C)** Nomogram composed of risk score, age, stage to predict 1-, 3-, 5-year OS probability. **(D)** Kaplan-Meier survival curves of OS between the high- and low-risk defined by nomogram. **(E)** Calibration curves of 1-, 3-, 5-year OS by nomogram. 1- **(F)**, 3- **(G)**, 5- **(H)** year ROC curves of age, gender, stage, treatment type, risk score, and nomogram. **(I)** DCA curves of age, gender, stage, treatment type, risk score, and nomogram.

Supplementary Figure 5 Single-cell transcriptomic profiles revealed the association of succinylation in LUAD and immunotherapy response. **(A)** The distribution of the succinylation scores in LUAD and normal lung tissues. LUAD displayed higher succinylation scores than normal lung tissues, especially in epithelial cells. **(B)** UMAP plot of the nine major cell populations form the NSCLC immunotherapy cohort (GSE207422). **(C)** Comparison of succinylation scores among the treatment-naïve (TN), major pathological responders (MPR) and non-responders (NMPR) groups. **(D)** UMAP plot shows the distribution of the succinylation scores among the TN, MPR and NMPR groups.

Supplementary Figure 6 GO enrichment and KEGG pathway analyses. GO enrichment analysis of upregulated genes in high-succinylation subtype **(A)** and upregulated genes in low-succinylation subtype **(B)**. KEGG pathway analyses of upregulated genes in high-succinylation subtype **(C)** and upregulated genes in low-succinylation subtype **(D)**.

Supplementary Figure 7 Intercellular communication pattern and the expression of ligand-receptor pairs. **(A)** The relative strength of each pathway in each cell type including the incoming and outgoing signaling patterns. The expression profiles of key ligand-receptor pairs in the collagen signaling pathways **(B)**, FN1 signaling pathways **(C)**, and APP signaling pathways **(D)**.

Supplementary Figure 8 Ligand-receptor pairs between malignant cells with different succinylation levels and other cell types. **(A)** Source cells: other cell types; Target cells: malignant cells with different succinylation levels. **(B)** Source cells: malignant cells with different succinylation levels; Target cells: other cell types.

Supplementary Figure 9 Identification of succinylation-related therapeutic targets in LUAD through high-dimensional weighted gene co-expression network analysis (hdWGCNA). **(A)** Gene dendrogram and module identification. Each color band below the dendrogram represents a co-expression module identified by dynamic tree cutting. **(B)** The average expression (color intensity) and percentage (dot size) of modular genes across three succinylation subclusters. **(C)** UMAP plots showing the expression patterns of the seven key genes across major cell types. **(D)** The differences of 7 candidate genes expression across three succinylation subclusters. **(E)** KLK6 mRNA expression is significantly upregulated in LUAD tissues compared to matched normal lung tissues. **(F)** Single-cell RNA-seq (scRNA-seq) analysis reveals KLK6 is predominantly expressed in malignant epithelial cells.

Supplementary Figure 10 Ligand-receptor pairs between malignant cells with different KLK6 expression and other cell types. **(A)** Source cells: malignant cells with different KLK6 expression; Target cells: other cell types. **(B)** Source cells: other cell types; Target cells: malignant cells with different KLK6 expression.

Supplementary Figure 11 The expression profiles of key ligand-receptor pairs. The expression profiles of key ligand-receptor pairs in the collagen signaling pathways **(A)**, FN1 signaling pathways **(B)**, MK signaling pathways **(C)**.
