# Supplementary material for: Succinylation heterogeneity in lung adenocarcinoma: from prognostic model to KLK6-driven tumor microenvironment remodeling
Source: Front Immunol. 2025 Nov 26;16:1718994. doi: 10.3389/fimmu.2025.1718994 (PMC12689508; doi:10.3389/fimmu.2025.1718994)
Supplement: Supplementary file 2 [file Table2.docx]

**Table S2. The sequence of primers in this study.**

| **Gene** | **Species** | **Forward primer** | **Reverse Primer** |
| --- | --- | --- | --- |
| KLK6 | Human | GGCCAGATCACCCAGAACAT | TCCTGGCTTCTCCTTTGATCC |
| ACTA2 | Human | GCTGGTGATGATGCTCCCA | GCCCATTCCAACCATTACTCC |
| FN1 | Human | CGGTGGCTGTCAGTCAAAG | AAACCTCGGCTTCCTCCATAA |
| COL1A1 | Human | GAGGGCCAAGACGAAGACATC | CAGATCACGTCATCGCACAAC |
| GAPDH | Human | CTTTGGTA TCGTGGAAGGACTC | GTAGAGGCAGG GATGATGTTCT |
