# Supplementary figures and images for: Succinylation heterogeneity in lung adenocarcinoma: from prognostic model to KLK6-driven tumor microenvironment remodeling
Source: Front Immunol. 2025 Nov 26;16:1718994. doi: 10.3389/fimmu.2025.1718994 (PMC12689508; doi:10.3389/fimmu.2025.1718994)

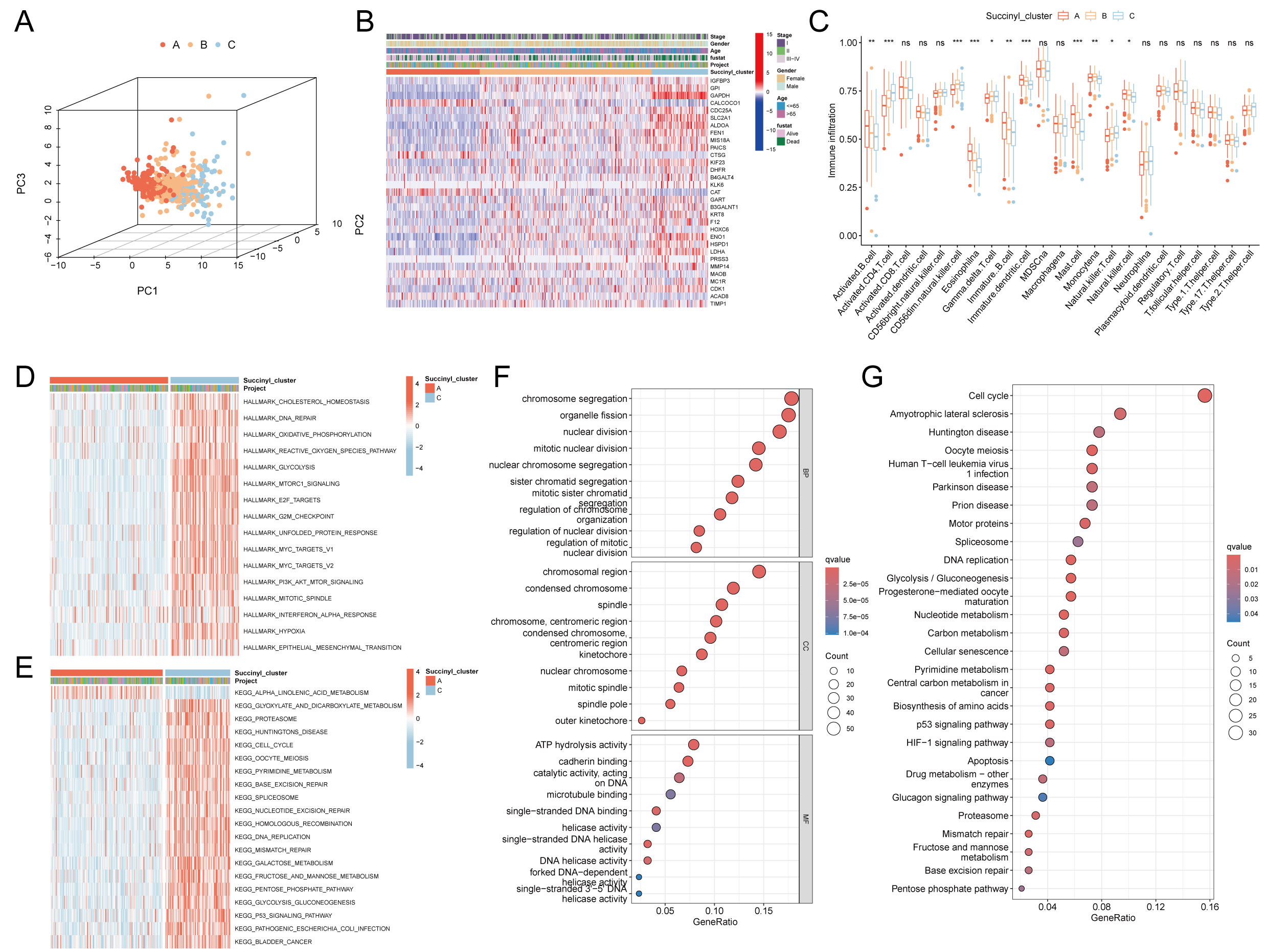

Supplement: Supplementary Figure 1 — Molecular subtyping of lung adenocarcinoma based on 31 core succinylation-related genes reveals distinct functional, and immune characteristics. (A) Three-dimensional PCA plot validating clear separation of the three identified molecular subtypes. (B) Heatmap integrating the expression patterns of the 31 core succinylation-related genes with key clinical-pathological features across the three subtypes. (C) Immune cell infiltration landscape assessed by ssGSEA among three subtypes. (D) Hallmark gene set analysis for Subtype C compared to subtype A. (E) KEGG pathway analysis for Subtype C compared to subtype A. (F) Gene Ontology (GO) analysis of common differentially expressed genes across all subtypes. (G) KEGG pathway analysis of common differentially expressed genes across all subtypes. PCA, principal component analysis; KEGG, Kyoto Encyclopedia of Genes and Genomes; GO, Gene Ontology; ssGSEA, single-sample gene set enrichment analysis. “*” means that p <0.05; “**” means that p < 0.01; “***” means that p < 0.001; ns, no significance. [file Image1.tif]

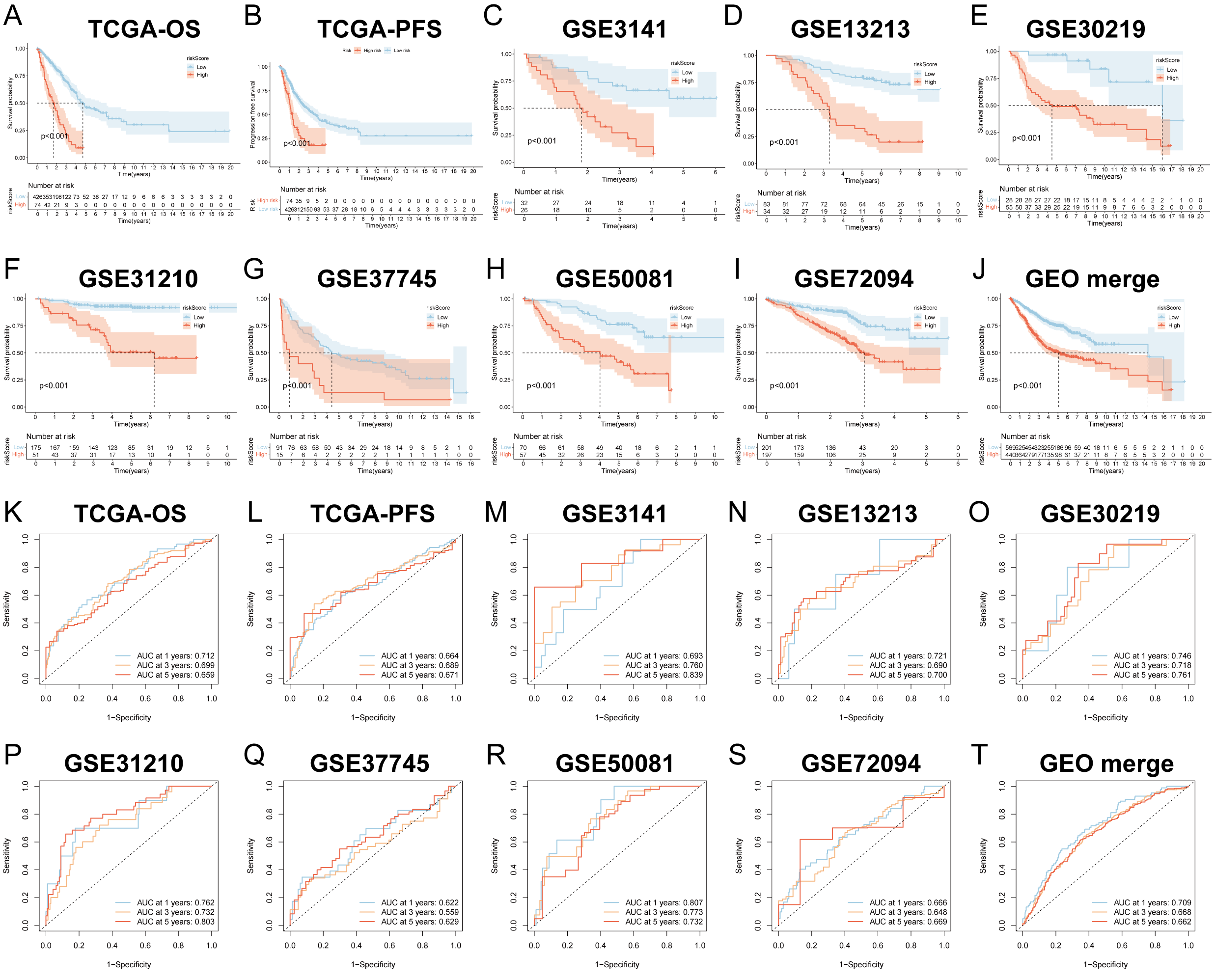

Supplement: Supplementary Figure 2 — Predictive performance of the succinylation-related model. Kaplan-Meier survival curves comparing OS and progression-free survival (PFS) between high-risk and low-risk groups across different cohorts. Patients in the high-risk group shows significantly poorer outcomes in the (A) TCGA-OS, (B) TCGA-PFS, (C) GSE3141, (D) GSE13213, (E) GSE30219, (F) GSE31210, (G) GSE37745, (H) GSE50081, (I) GSE72094, and (J) merged GEO datasets (Log-rank test, all p< 0.001). Time-dependent receiver operating characteristic (ROC) curves assessing the predictive accuracy of the model for 1-, 3-, and 5-year survival. The area under the curve (AUC) values for each time point are indicated in the respective cohorts: (K) TCGA-OS, (L) TCGA-PFS, (M) GSE3141, (N) GSE13213, (O) GSE30219, (P) GSE31210, (Q) GSE37745, (R) GSE50081, (S) GSE72094, and (T) GEO merge. [file Image2.tif]

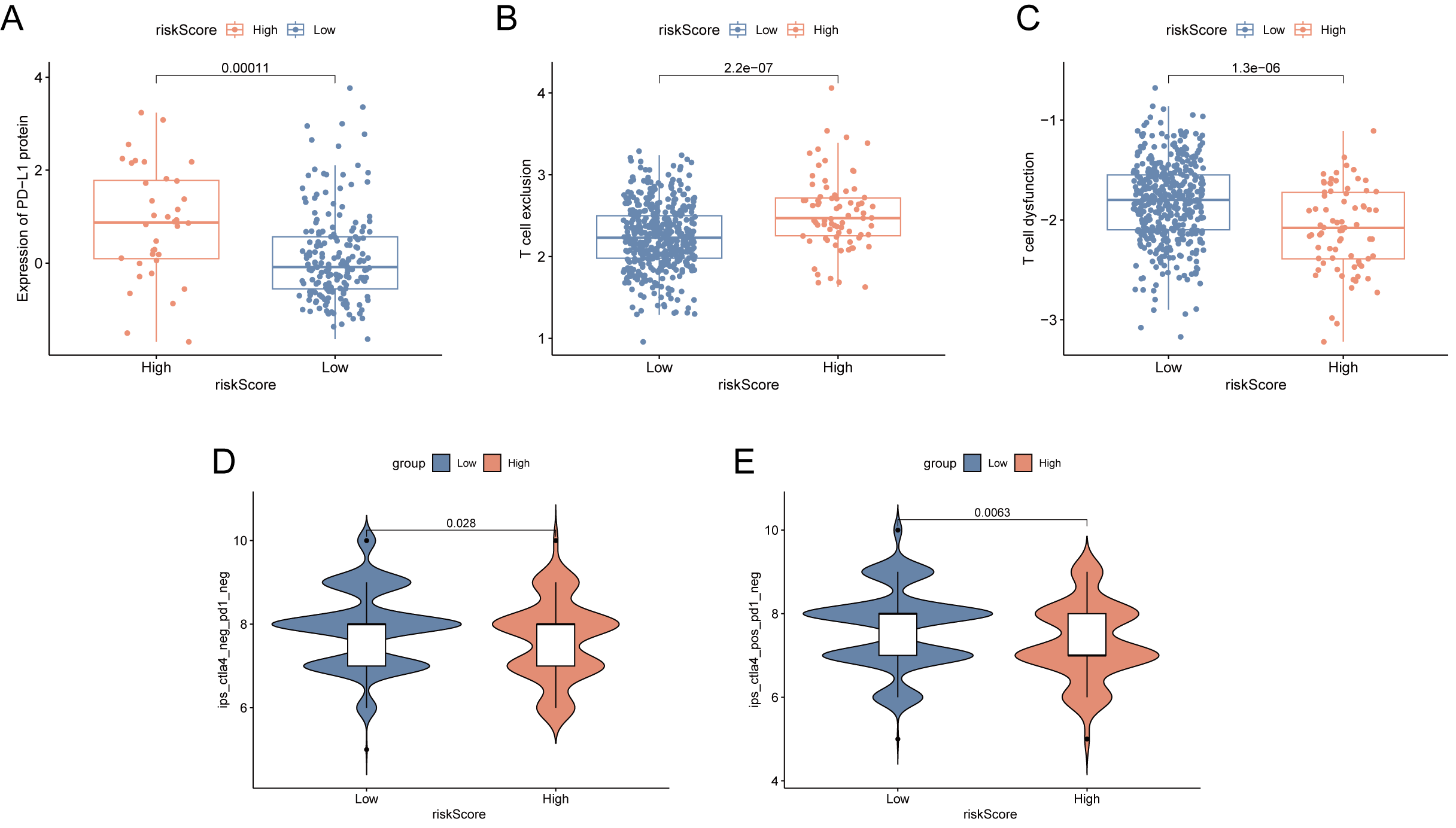

Supplement: Supplementary Figure 3 — TIDE score and IPS difference between low- and high-risk LUAD groups. (A) PD-L1 protein expression was elevated in the high-risk group than the low-risk group. LUAD patients in the high-risk group had higher T cell exclusion (B) but lower T cell dysfunction scores (C) from the TIDE analysis. LUAD patients in the low-risk group had higher IPS with CTLA4- and PD-1- (D), CTLA4+ and PD-1- (E). [file Image3.tif]

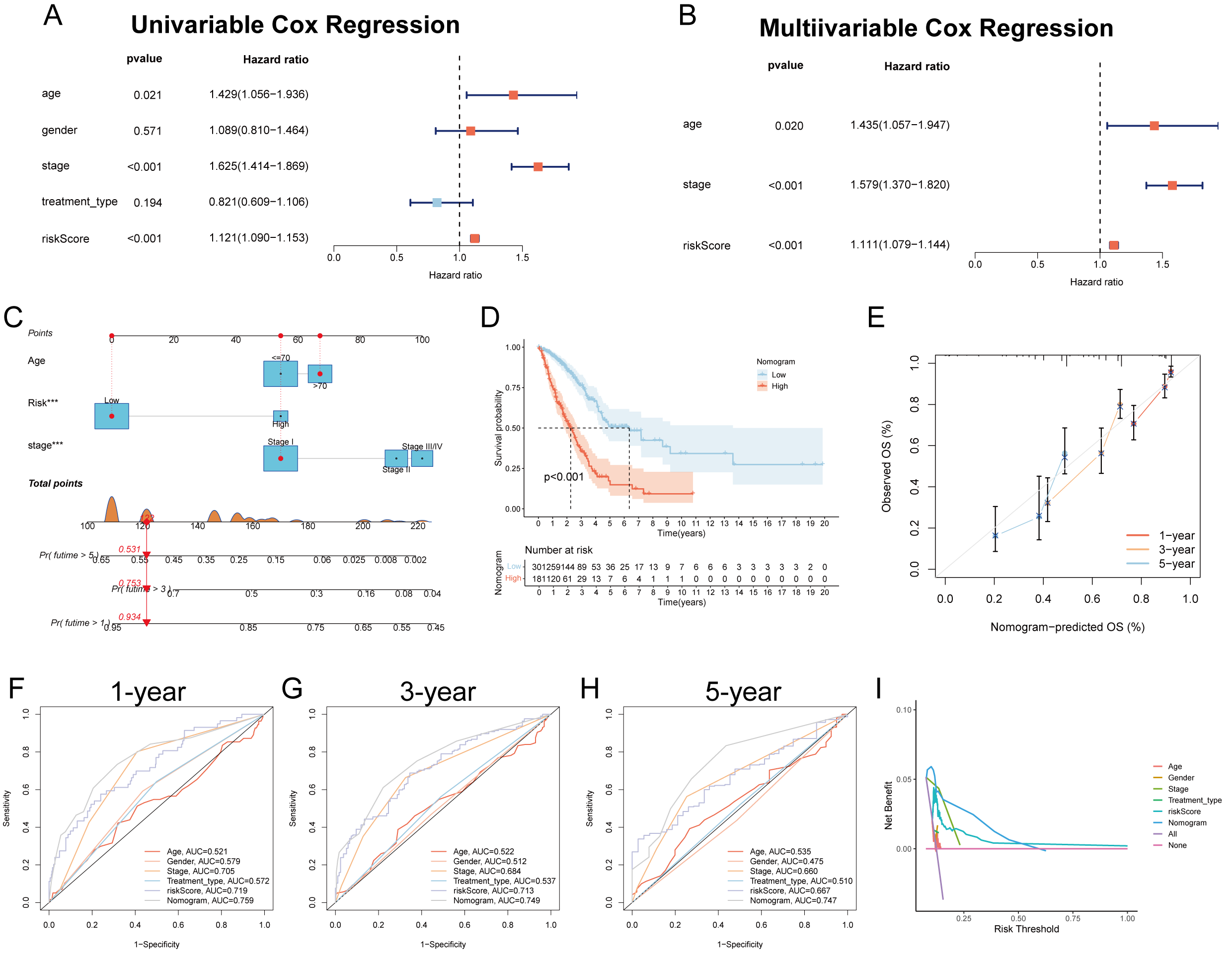

Supplement: Supplementary Figure 4 — Development and evaluation of a prognostic nomogram. Univariable (A) and multivariable (B) Cox regression analyses of risk score and clinical features. (C) Nomogram composed of risk score, age, stage to predict 1-, 3-, 5-year OS probability. (D) Kaplan-Meier survival curves of OS between the high- and low-risk defined by nomogram. (E) Calibration curves of 1-, 3-, 5-year OS by nomogram. 1- (F), 3- (G), 5- (H) year ROC curves of age, gender, stage, treatment type, risk score, and nomogram. (I) DCA curves of age, gender, stage, treatment type, risk score, and nomogram. [file Image4.tif]

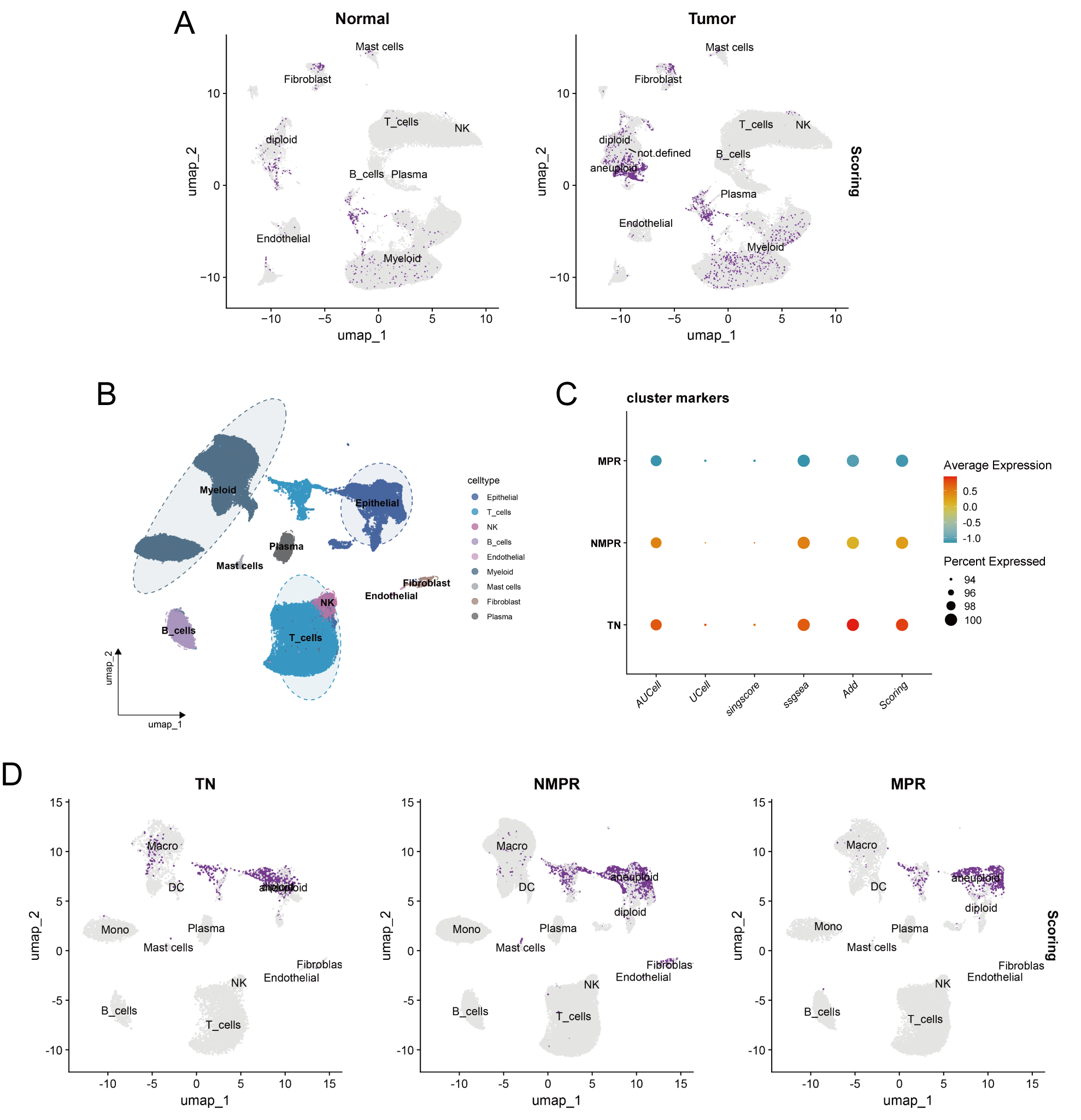

Supplement: Supplementary Figure 5 — Single-cell transcriptomic profiles revealed the association of succinylation in LUAD and immunotherapy response. (A) The distribution of the succinylation scores in LUAD and normal lung tissues. LUAD displayed higher succinylation scores than normal lung tissues, especially in epithelial cells. (B) UMAP plot of the nine major cell populations form the NSCLC immunotherapy cohort (GSE207422). (C) Comparison of succinylation scores among the treatment-naïve (TN), major pathological responders (MPR) and non-responders (NMPR) groups. (D) UMAP plot shows the distribution of the succinylation scores among the TN, MPR and NMPR groups. [file Image5.tif]

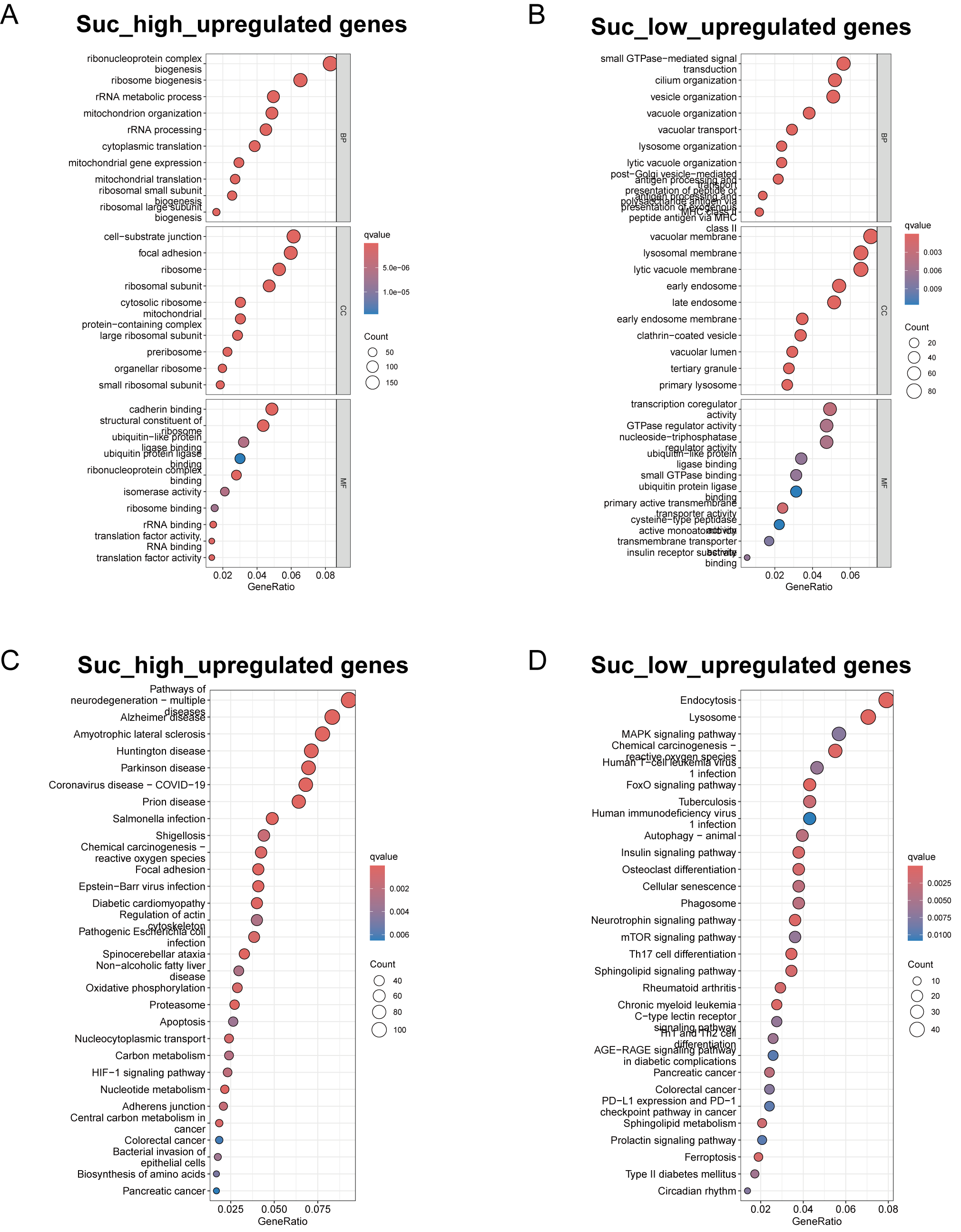

Supplement: Supplementary Figure 6 — GO enrichment and KEGG pathway analyses. GO enrichment analysis of upregulated genes in high-succinylation subtype (A) and upregulated genes in low-succinylation subtype (B). KEGG pathway analyses of upregulated genes in high-succinylation subtype (C) and upregulated genes in low-succinylation subtype (D). [file Image6.tif]

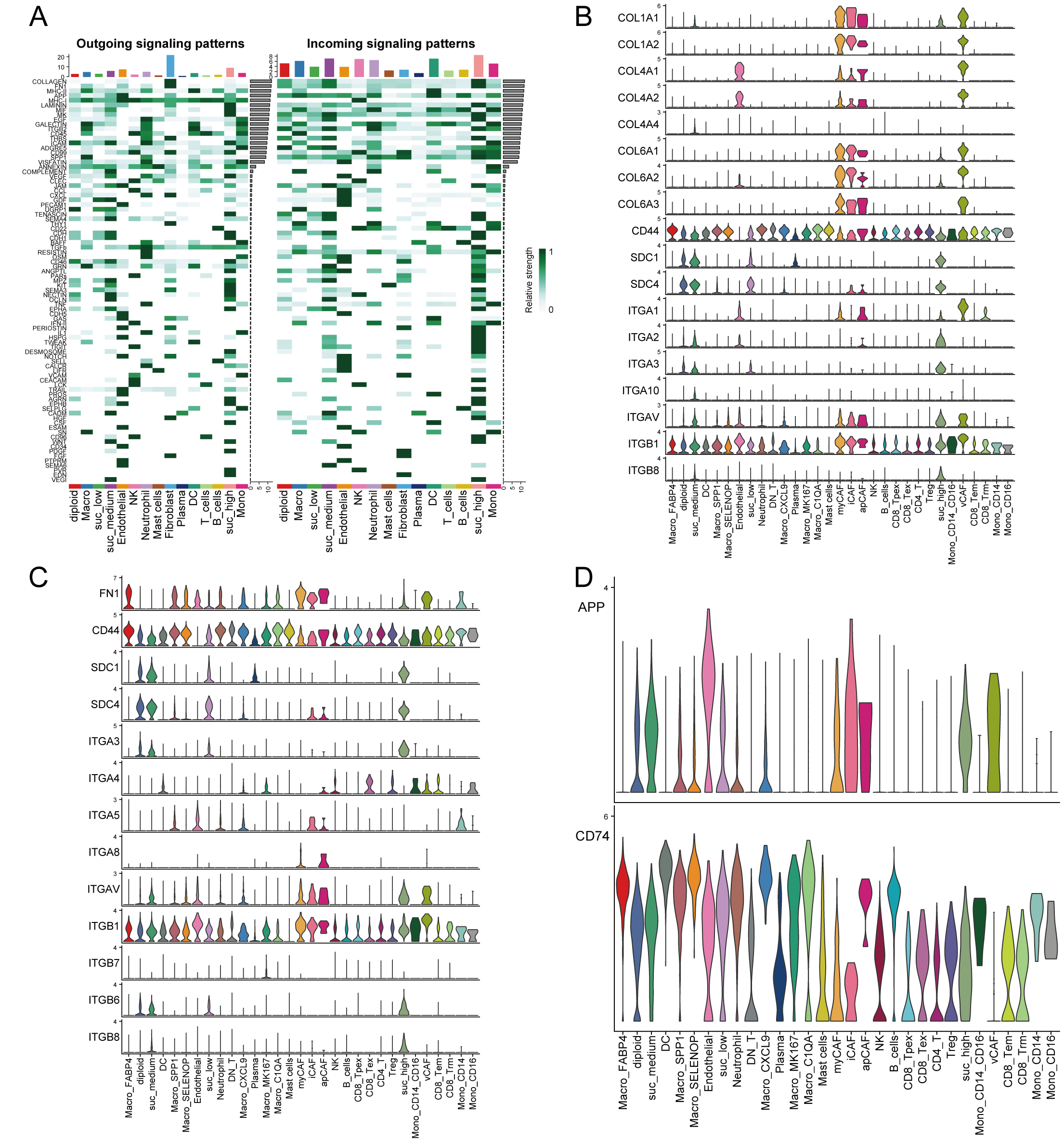

Supplement: Supplementary Figure 7 — Intercellular communication pattern and the expression of ligand-receptor pairs. (A) The relative strength of each pathway in each cell type including the incoming and outgoing signaling patterns. The expression profiles of key ligand-receptor pairs in the collagen signaling pathways (B), FN1 signaling pathways (C), and APP signaling pathways (D). [file Image7.tif]

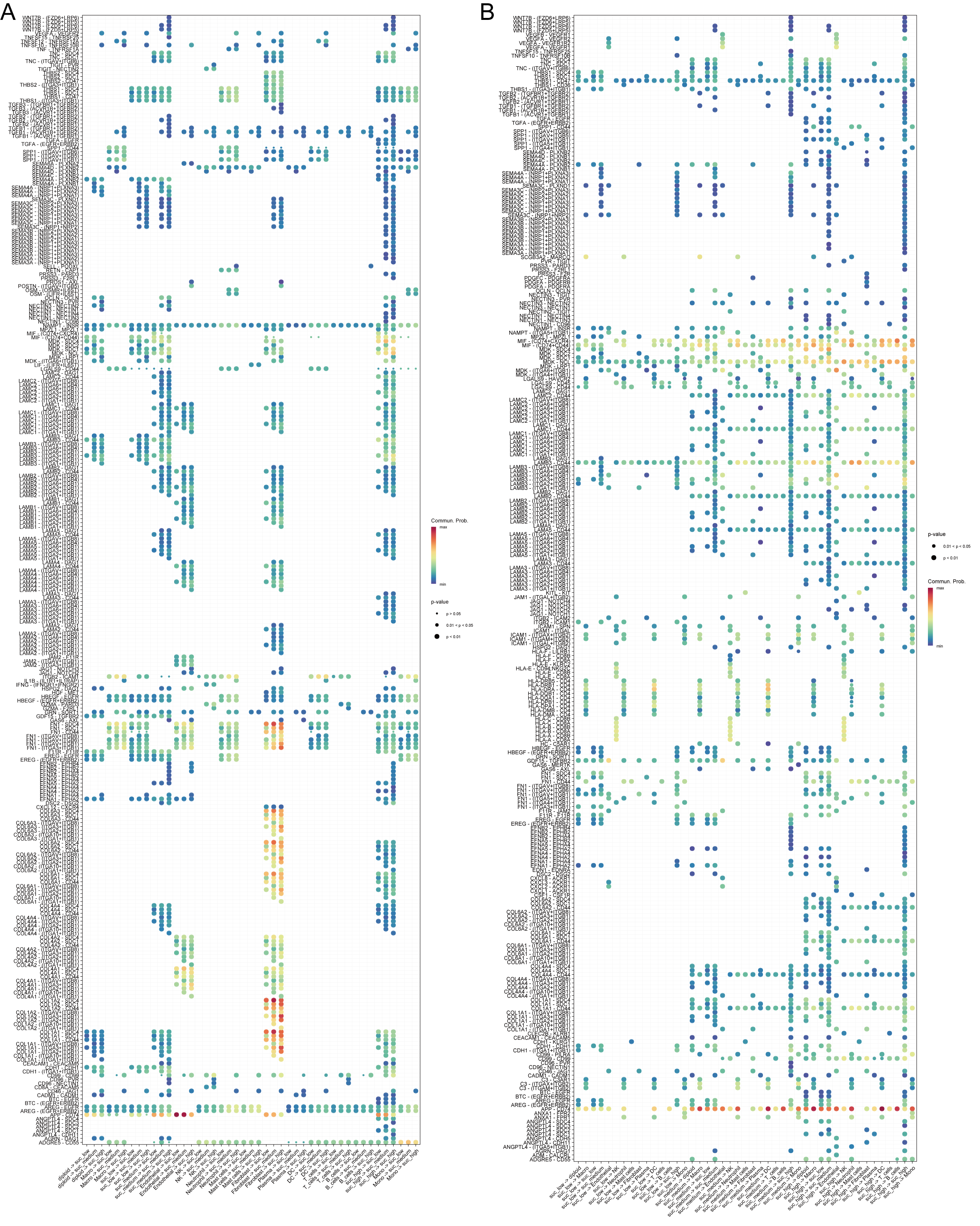

Supplement: Supplementary Figure 8 — Ligand-receptor pairs between malignant cells with different succinylation levels and other cell types. (A) Source cells: other cell types; Target cells: malignant cells with different succinylation levels. (B) Source cells: malignant cells with different succinylation levels; Target cells: other cell types. [file Image8.tif]

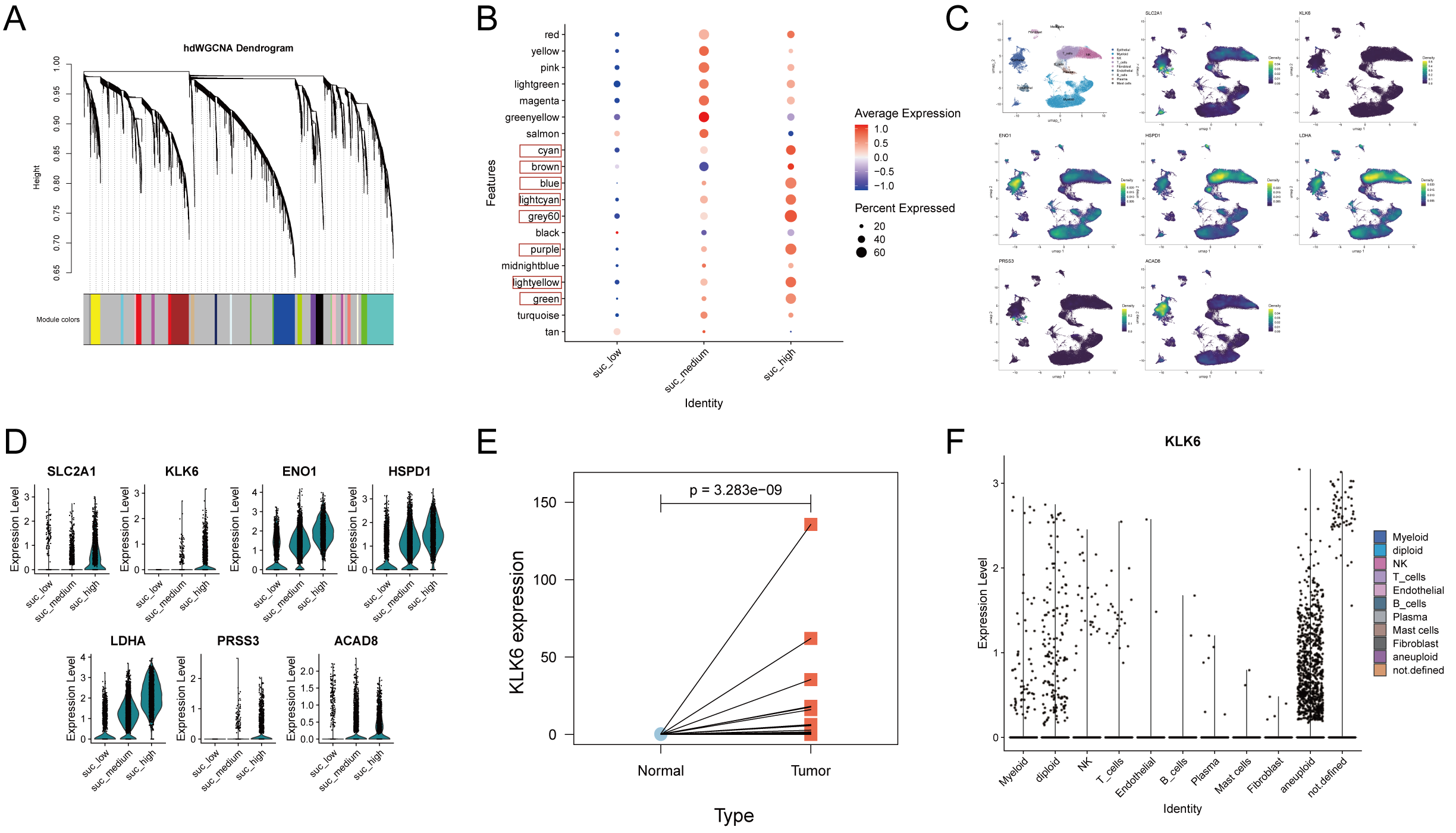

Supplement: Supplementary Figure 9 — Identification of succinylation-related therapeutic targets in LUAD through high-dimensional weighted gene co-expression network analysis (hdWGCNA). (A) Gene dendrogram and module identification. Each color band below the dendrogram represents a co-expression module identified by dynamic tree cutting. (B) The average expression (color intensity) and percentage (dot size) of modular genes across three succinylation subclusters. (C) UMAP plots showing the expression patterns of the seven key genes across major cell types. (D) The differences of 7 candidate genes expression across three succinylation subclusters. (E) KLK6 mRNA expression is significantly upregulated in LUAD tissues compared to matched normal lung tissues. (F) Single-cell RNA-seq (scRNA-seq) analysis reveals KLK6 is predominantly expressed in malignant epithelial cells. [file Image9.tif]

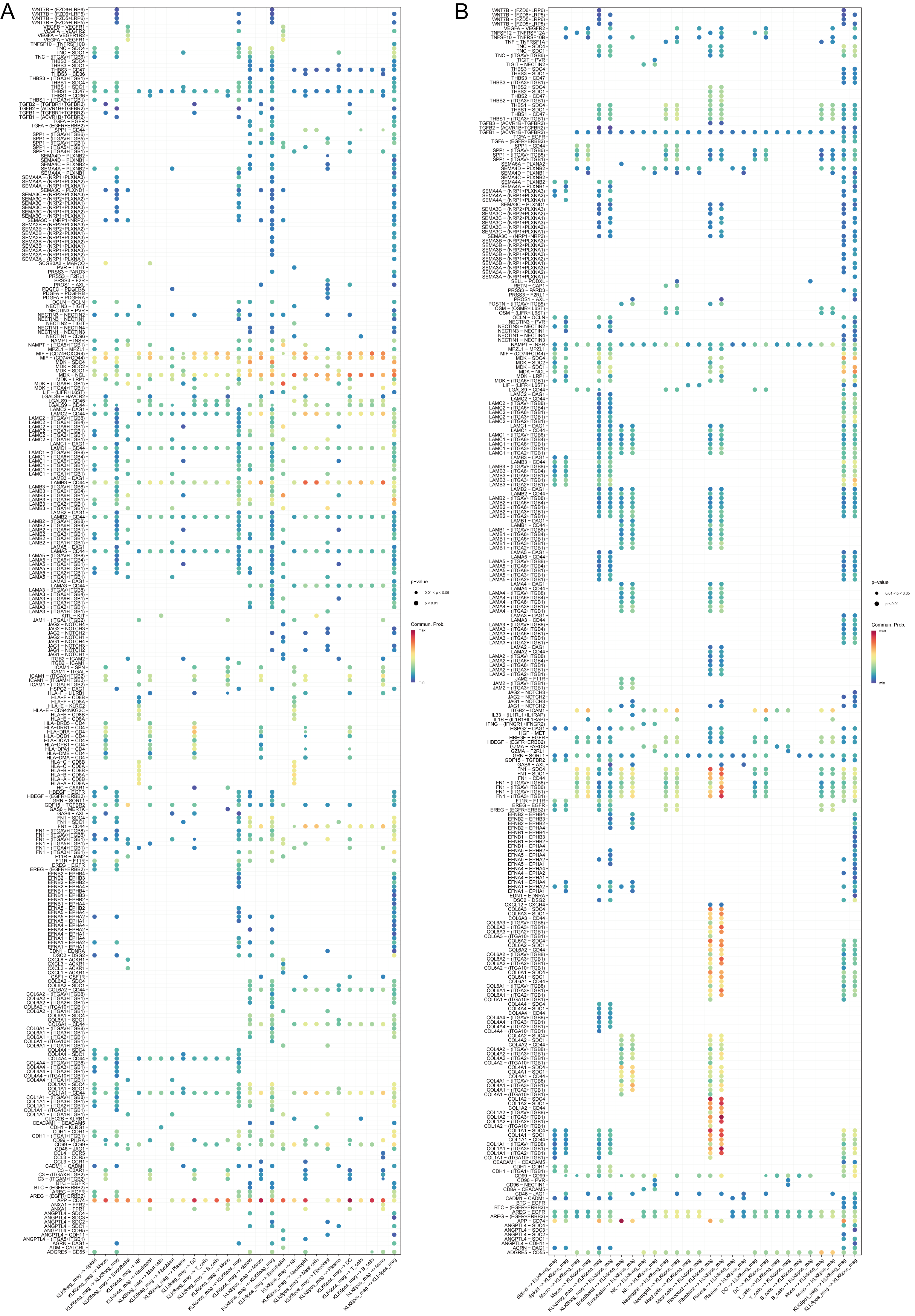

Supplement: Supplementary Figure 10 — Ligand-receptor pairs between malignant cells with different KLK6 expression and other cell types. (A) Source cells: malignant cells with different KLK6 expression; Target cells: other cell types. (B) Source cells: other cell types; Target cells: malignant cells with different KLK6 expression. [file Image10.tif]

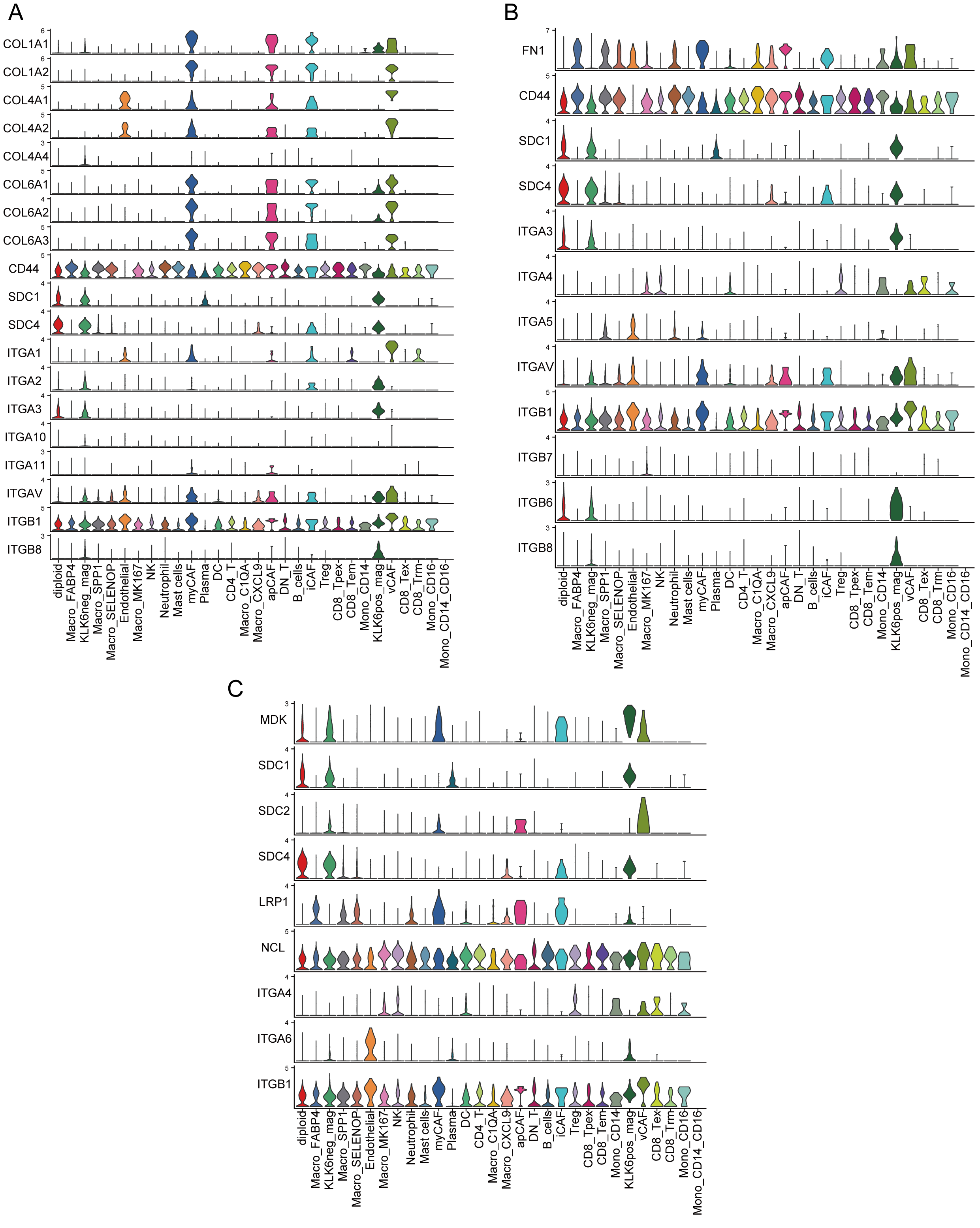

Supplement: Supplementary Figure 11 — The expression profiles of key ligand-receptor pairs. The expression profiles of key ligand-receptor pairs in the collagen signaling pathways (A), FN1 signaling pathways (B), MK signaling pathways (C). [file Image11.tif]
